# Environmentally-acquired bacteria influence microbial diversity and natural innate immune responses at gut surfaces

**DOI:** 10.1186/1741-7007-7-79

**Published:** 2009-11-20

**Authors:** Imke E Mulder, Bettina Schmidt, Christopher R Stokes, Marie Lewis, Mick Bailey, Rustam I Aminov, James I Prosser, Bhupinder P Gill, John R Pluske, Claus-Dieter Mayer, Corran C Musk, Denise Kelly

**Affiliations:** 1 Gut Immunology Group, University of Aberdeen, Rowett Institute of Nutrition and Health, Greenburn Road, Aberdeen AB21 9SB, UK; 2 Veterinary Pathology, Infection & Immunity, Langford House, Langford, Bristol, BS40 5DU, UK; 3 Institute of Biological and Environmental Sciences, University of Aberdeen, St Machar Drive, Aberdeen AB24 3UU, UK; 4 Agricultural and Horticultural Development Board, Winterhill House, Snowdon Drive, Milton Keynes MK6 1AX, UK; 5 School of Veterinary and Biomedical Sciences, Murdoch University, Murdoch, WA 6150, Australia; 6 Biomathematics & Statistics Scotland, University of Aberdeen, Rowett Institute of Nutrition and Health, Greenburn Road, Aberdeen AB21 9SB, UK

## Abstract

**Background:**

Early microbial colonization of the gut reduces the incidence of infectious, inflammatory and autoimmune diseases. Recent population studies reveal that childhood hygiene is a significant risk factor for development of inflammatory bowel disease, thereby reinforcing the hygiene hypothesis and the potential importance of microbial colonization during early life. The extent to which early-life environment impacts on microbial diversity of the adult gut and subsequent immune processes has not been comprehensively investigated thus far. We addressed this important question using the pig as a model to evaluate the impact of early-life environment on microbe/host gut interactions during development.

**Results:**

Genetically-related piglets were housed in either indoor or outdoor environments or in experimental isolators. Analysis of over 3,000 16S rRNA sequences revealed major differences in mucosa-adherent microbial diversity in the ileum of adult pigs attributable to differences in early-life environment. Pigs housed in a natural outdoor environment showed a dominance of Firmicutes, in particular *Lactobacillus*, whereas animals housed in a hygienic indoor environment had reduced *Lactobacillus *and higher numbers of potentially pathogenic phylotypes. Our analysis revealed a strong negative correlation between the abundance of Firmicutes and pathogenic bacterial populations in the gut. These differences were exaggerated in animals housed in experimental isolators. Affymetrix microarray technology and Real-time Polymerase Chain Reaction revealed significant gut-specific gene responses also related to early-life environment. Significantly, indoor-housed pigs displayed increased expression of Type 1 interferon genes, Major Histocompatibility Complex class I and several chemokines. Gene Ontology and pathway analysis further confirmed these results.

**Conclusion:**

Early-life environment significantly affects both microbial composition of the adult gut and mucosal innate immune function. We observed that a microbiota dominated by lactobacilli may function to maintain mucosal immune homeostasis and limit pathogen colonization.

## Background

The gastrointestinal tract contains an immense number of micro-organisms, collectively known as the microbiota. The major functions of the microbiota include degrading dietary compounds, influencing nutrient partitioning and lipid metabolism, providing essential nutrients generated as a result of microbial metabolism, protecting against invading pathogens and stimulating gut morphology [1-4]. The gut microbiota also plays an important role in maintaining immune function. Recent work suggests that the commensal microbiota influences processes as complex as pathogen colonization, immune development and homeostasis, T cell differentiation, inflammation, repair and angiogenesis [5-8].

The impact of the microbiota on host immunity is thought to be critically regulated in early life and *inappropriate exposure *to bacteria during this developmental window has been linked to the increased incidence of infectious, inflammatory and autoimmune diseases [9-11]. Clearly, the neonatal period is a critical time for gut colonization, and can be affected by numerous factors including gestational age, birth environment, mode of delivery, nutrition and antibiotic use [12,13].

The increase in immune-mediated disorders, particularly in Westernized countries, has led to the so-called *Hygiene Hypothesis*, which postulates that the growing incidence of immune-mediated diseases is the consequence of reduced infection and exposure to microbes during early childhood [14]. In this context, the high-hygiene status of western lifestyle, decreased infection rates and reduced bacterial load as a result of widespread use of vaccines and antibiotics are likely to be important contributory factors [15]. Animal models have provided some insight into immune-disease aetiology: animals susceptible to autoimmune disease have an increased incidence and severity of disease when bred under germ-free conditions whereas disease is prevented when the animals are exposed to bacteria [16]. This evidence supports the notion that, in addition to naturally-acquired infections, colonization by the normal commensal microbiota is an important factor limiting the incidences of immune-mediated diseases. Consistent with this is the growing awareness of the importance of the commensal microbiota in immune education in early life [8], which appears to involve complex mechanisms of host-bacterial crosstalk [5,17-21].

In the current study we have investigated potential interactions between the rearing environment, gut microbiota and immune function in the developing pig gut using molecular methods to evaluate both microbial diversity and host immune gene expression. Microbial diversity in the gastrointestinal tract of these animals was characterized by sequence analysis of 16S rRNA gene libraries. Specific responses in transcriptome expression patterns of gut ileal tissue were studied using Affymetrix GeneChip Porcine Genome microarrays (Affymetrix, Santa Clara, CA). Biomarkers associated with immune function and altered by rearing environment were identified and investigated more thoroughly by Real-time Polymerase Chain Reaction (PCR).

## Results

### Mucosal microbial diversity in the ileum of pigs from different environments

We investigated the influence of environmentally-acquired bacteria on the composition of the adult mucosa-adherent ileal microbiota in the pig. Animals were housed in an indoor (IN) or an outdoor facility (OUT), as well as in individual isolator units receiving daily doses of antibiotics (IR). Mucosa-adherent bacterial samples from the ileum and fecal samples were collected from all experimental animals at day 56. In addition to this, fecal samples were taken from adult sows from both the indoor (INS) and outdoor (OUTS) environments to confirm 'environment' as the major factor contributing to the experimental differences. Microbial composition of the ileum was examined by calculation of diversity indices and analysis of the phylogenetic distribution of 16S rRNA gene sequences derived from clone libraries of each treatment. After quality control, a total of 3,089 validated clones were analyzed.

### Diversity Measures

**Figure 1:**
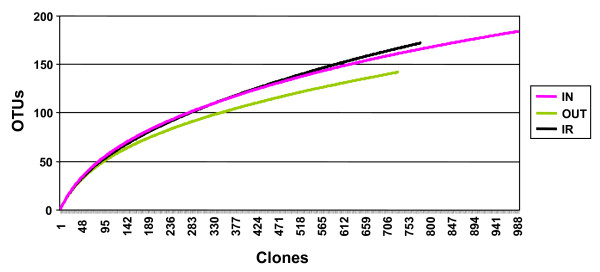
**Treatment-based rarefaction curves of 16S rRNA gene libraries**. The rarefaction curves from IN (pink), OUT (green) and IR (black) animals were generated by plotting the number of phylotypes (OTUs; defined at 99% sequence identity) against the number of clones sequenced. The shape of the curves of observed phylotype richness indicates a trend of diminishing chance of finding new phylotypes as sampling continues.

We first investigated the effects of environment and high-hygiene status on a number of bacterial diversity indices. Estimates of diversity, richness and library coverage for the 16S rRNA clone libraries from IN, OUT and IR are shown in Table 1. Species richness, estimated by Chao1, was highest in the IR and IN groups but lower in the OUT group. Good's coverage was 90.97 to 93.47% for all three treatment groups, with the lowest coverage in IR libraries. Rarefaction analysis of clone libraries confirmed these findings and suggested that the IR and IN groups possessed the most diverse mucosa-adherent bacterial community, whereas the OUT group showed lower microbial diversity (Figure 1).

**Table 1:** Indices of diversity, richness and library coverage for 16S rRNA gene libraries.

Measurement	IN	OUT	IR
**No. of clones**	995	734	780
**Chao1 estimator of species richness**	259.02	254.89	302.53
**Shannon diversity index (H)**	4.438	4.190	4.355
**Simpson diversity index (1-D)**	0.980	0.975	0.979
**Simpson reciprocal index (1/D)**	52.00	40.41	47.47

**Good's estimator of coverage (%)**	93.47	92.46	90.97

Calculations were made based on OTU definition at 99% sequence identity (N = 4).

**Figure 2:**
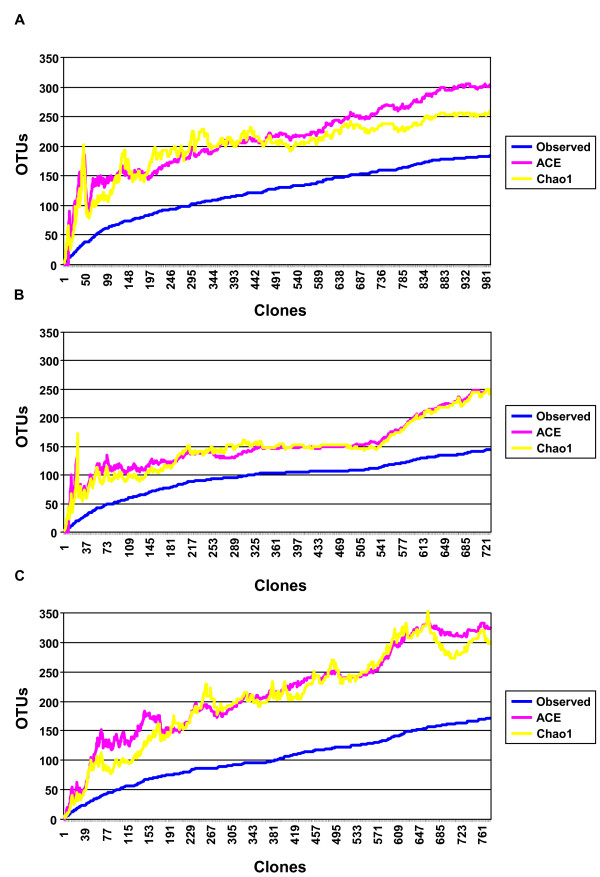
**Collector's curves of the observed and estimated phylotype richness of 16S rRNA gene libraries**. Collector's curves of the observed (blue) and estimated (ACE (pink) and Chao1 (yellow)) phylotype richness calculated for IN **(A)**, OUT **(B) **and IR **(C) **at 99% level. Each curve reflects observed or estimated richness values obtained as more clones are added to the data set. After an initial steep rise, the curves level out suggesting that a majority of clones in the treatment groups have been sampled. Differences between the estimates and observed phylotype richness were highest in the IR group. Novel phylotypes continued to be identified up to the end of sampling in this group.

Collector's curves of the observed and estimated phylotype richness are shown in Figure 2A-C. Each curve reflects the series of observed or estimated richness values obtained as more clones were added to the data set. After an initial steep rise, the curves level out, suggesting that the majority of phylotypes in the treatment groups were adequately sampled. In the early stages of sampling and clone sequencing, both Chao1 and abundance-based coverage estimator (ACE) showed a sharp increase, together with the observed phylotype number, in the IN group (Figure 2A). After the sampling of about 190 clones, the gap between the observed and estimated phylotype richness was relatively constant, indicating repeated sampling of same phylotypes within samples. In the OUT group, the gap between the observed and estimated phylotype richness was constant after the sampling point of 110 clones (Figure 2B). The difference between the estimated and observed phylotype richness was highest in the IR mucosal libraries. Novel phylotypes continued to be identified up to the end of sampling (Figure 2C).

### Phylogenetic affiliation of 16S rRNA gene sequences

**Figure 3:**
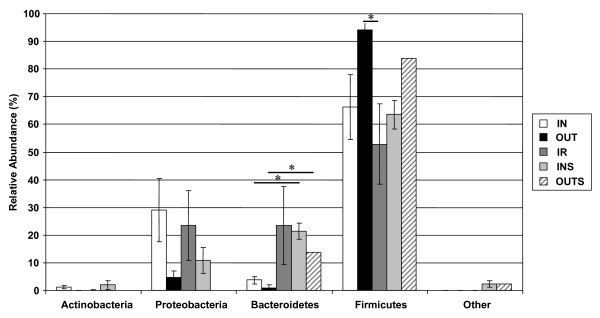
**Phylogenetic distribution of clones obtained from mucosa-adherent ileal and fecal samples in different housing environments**. The majority of clones were assigned to the phyla Firmicutes, Proteobacteria, Bacteroidetes, and Actinobacteria. The Firmicutes phylum was significantly increased in the OUT group compared to the IR group (*P *< 0.05). The Bacteroides phylum was significantly increased in both the INS and OUTS fecal libraries compared to the mucosa-adherent ileal libraries. Values are expressed as means ± SEM (N = 4).

Phylogenetic analysis was performed to establish taxonomic positioning of obtained sequences. All 16S rRNA gene sequences from the mucosa-adherent ileal and fecal samples were subjected to the Ribosomal Database Project (RDP) Classifier analysis (95% confidence threshold). Based on the classification results, the majority of clones were assigned to four phyla: Firmicutes (69.7% of all sequences), Proteobacteria (17.7%), Bacteroidetes (11.4%), and Actinobacteria (0.5%) (Figure 3 and Table 2). The two major phyla, Firmicutes and Bacteroidetes, were significantly different between the libraries: the Firmicutes showed a significant increase in the OUT group compared to the IR group, while the Bacteroidetes were significantly increased in both the INS and OUTS fecal libraries compared to the mucosa-adherent ileal libraries.

**Table 2:** Major phylogenetic distribution of 16S rRNA gene sequences and phylotypes from treatment groups.

Phylum	Bacterial taxa	IN	OUT	IR	INS	OUTS
**% Bacteroidetes**		**3.72**	**1.08**	**20.97**	**26.44**	**13.70**
	Prevotellaceae (%)	2.91	0.54	18.79	10.09	4.83
	Bacteroidaceae (%)	0.40	0	0.76	4.08	0
	Porphyromonadaceae (%)	0	0.40	0.63	3.84	8.87
						
**% Proteobacteria**		**28.26**	**4.63**	**24.04**	**12.25**	**0**
	**α-proteobacteria (%)**	**0**	**0.13**	**0**	**0**	**0**
	**β-proteobacteria (%)**	**0.20**	**0**	**0.1**	**0.24**	**0**
	**γ-proteobacteria (%)**	**15.19**	**3.81**	**23.91**	**11.53**	**0**
	Pasteurellaceae (%)	14.78	2.17	2.55	0	0
	Enterobacteriaceae (%)	0.4	1.36	20.7	0.24	0
	Pseudomonadaceae (%)	0	0	0	4.08	0
	Moraxellaceae (%)	0	0	0.63	6.25	0
	**ε-proteobacteria (%)**	**12.97**	**0.4**	**0**	**0.48**	**0**
	Helicobacteraceae (%)	10.46	0.4	0	0	0
	Campylobacteraceae (%)	2.61	0	0	0.48	0
						
**% Firmicutes**		**66.29**	**94.0**	**54.21**	**73.55**	**83.06**
	**Erysipelotrichi (%)**	**0.90**	**0**	**0.12**	**0.72**	**0**
	**Bacilli (%)**	**18.81**	**81.8**	**3.70**	**19.95**	**49.18**
	Order Bacillales (%)	0.2	0.2	0.12	5.28	0
	Order Lactobacillales (%)	18.6	81.6	3.58	14.42	49.18
	**Clostridia (%)**	**46.68**	**12.12**	**47.69**	**51.92**	**33.87**
	Lachnospiraceae (%)	3.52	0.95	8.95	3.84	8.87
	Veillonellaceae (%)	0	0.4	6.9	1.68	1.61
	Clostridiaceae (%)	13.17	2.72	1.27	19.95	8.06
	Peptostreptococcaceae (%)	24.44	7.49	28.9	15.38	2.41
	Ruminococcaceae (%)	0.90	0.54	6.26	6.97	7.25

### Firmicutes

Seventy percent of all sequences were affiliated with the Firmicutes phylum. The outdoor environment favoured the expansion of Firmicutes compared to the hygienic environment (Figure 3). At the lower taxa level this difference was even more pronounced.

A large number of sequenced clones fell into the Bacilli class. The most abundant order was Lactobacillales, which was dominated by Lactobacillaceae, but also contained Streptococcaceae, Leuconostocaceae, Enterococcaceae, Carnobacteriaceae and Aerococcaceae, although present in lower abundance.

**Figure 4:**
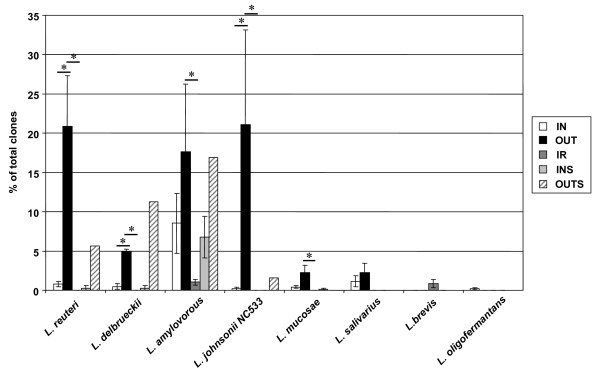
**Abundance of lactobacilli in mucosa-adherent ileal and fecal samples in different housing environments**. The Lactobacillaceae family included *L. reuteri*, *L. amylovorous *LAB31, *L. johnsonii*, *L. delbrueckii *subsp. *bulgaricus*, *L salivarius *and *L. mucosae. L. reuteri*, *L. delbrueckii *and *L. johnsonii *were all significantly lower in the IN and IR groups compared to the OUT group. Values are expressed as means ± SEM (N = 4).

The Lactobacillaceae family in the OUT group (77.2% of sequences) consisted of a small number of operational taxonomic units (OTUs), including *Lactobacillus reuteri*, *L. amylovorous *LAB31, *L. johnsonii*, *L. delbrueckii *subsp. *bulgaricus*, *L. salivarius *and *L. mucosae *(Figure 4). In contrast, the IN library contained only 12.8% Lactobacillaceae-affiliated clones, although phylotypes were similar to those observed in the OUT group. *L. reuteri*, *L. delbrueckii *and *L. johnsonii *were all significantly decreased compared to the OUT group. The high-hygiene conditions associated with IR exacerbated these differences. *L. amylovorous *LAB31 and *L. brevis *were present in very low abundance in the IR libraries (3.58% of sequences) whereas *L. reuteri, L. delbrueckii *subsp. *bulgaricus, L. johnsonii *and *L. mucosae *were not detected in this treatment group.

The observed differences in *Lactobacillus *levels between the IR and OUT group were confirmed by enumeration of bacteria in gut contents of both ileum and colon on de Man, Rogosa and Sharpe (MRS) agar. The OUT group had three to four log_10 _colony forming units lactobacilli/g more than the IR group, thus validating the 16S rRNA gene library results (Additional file 1).

Members of the Clostridia class were present in all treatment groups with 28.2% of all sequenced clones classified as Clostridia. Interestingly, pigs raised in the indoor environment showed the highest abundance of this class.

**Figure 5:**
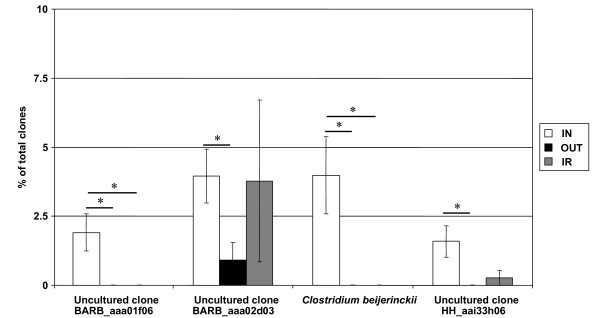
**Significantly affected bacterial clones in the mucosa-adherent ileum of animals in different housing environments**. *Clostridium beijerinckii *NCIMB 8052, as well as uncultured bacterial clone BARB_aaa01f06, BARB_aaa02d03 and HH_aai33h06 were significantly decreased in the OUT library compared to the IN library. Uncultured bacterial clone BARB_aaa01f06 and *Clostridium beijerinckii *NCIMB 8052 were also significantly decreased in the IR group. Values are expressed as means ± SEM (N = 4).

Clostridiaceae-affiliated clones were highly abundant in the IN group and mainly identified as uncultured species. *Clostridium beijerinckii *NCIMB 8052 was significantly elevated in the IN libraries compared to the OUT and IR libraries (Figure 5). The indoor environment also favoured the expansion of the bacterial clone HH_aai33h06 (EU775688) on the ileal mucosa compared to the outdoor environment.

The Peptostreptoccocaceae family was another abundant member of the Clostridia class, accounting for 19.1% of the sequenced clones in all treatment groups. The indoor environment favoured the expansion of Peptostreptoccocaceae. Seven predominant OTUs, represented by mainly uncultured clones, were identified. Sequences of uncultured clones BARB_aaa02d03 were significantly higher in the IN and IR groups compared to the OUT group and were not detected in the indoor fecal libraries (Figure 5). This possibly points to a preferential colonization of the ileal mucosa in the indoor environment. Uncultured bacterium clone BARB_aaa01f06 was significantly increased in the ileal mucosal libraries of the IN group compared to the OUT and IR groups, indicating a potential antibiotic sensitivity of this bacterium.

### Bacteroidetes

Bacteroidetes were found in all libraries but in different abundance. The most abundant group within this phylum was represented by members of the Prevotellaceae family, followed by Porphyromonadaceae, Bacteroidaceae and, to a lesser extent, Sphingobacteriaceae and Flavobacteriaceae.

All 16S rRNA gene libraries contained members of Prevotellaceae, yet they were most prevalent in the indoor environment, particularly in fecal libraries. High-hygiene conditions increased the numbers of Prevotellaceae on the ileal mucosa.

Porphyromonadaceae were mainly obtained from the fecal libraries of both farms. Most clones had only 97% similarity to previously isolated clones, specifically the Porphyromonadaceae bacterium sp DJF_B175 (EU728718) and uncultured bacterial clones (EU472597, EU472617 and EU461958).

Bacteroidaceae were exclusively obtained from the indoor environment. Within the IN and IR groups, these included *Bacteroides vulgatus *(CP000139) and uncultured bacterial clones (EF403095, EF403812, EU779318 and DQ800210). In the IN fecal libraries two species were related to *B. propionifaciens *(AB264625.2) and uncultured bacterium clone p-240-o5 (AF371909).

### Proteobacteria

Eighteen percent of all clones were placed into the Proteobacteria phylum. γ-proteobacteria and ε-proteobacteria were the most abundant groups, while members of the α- and β-proteobacteria were found only sporadically.

Twenty-eight γ-proteobacteria clones were obtained from the OUT mucosa-adherent libraries. These included *E. coli*, *Actinobacillus minor *and *A. porcinus*. Six OTUs belonging to *Actinobacillus *spp. were predominately present in the IN group, including *Actinobacillus minor*, *A. porcinus *strains H1498/H1215 and *A. rossii *strain JF1390. This clone has been isolated from the intestine and reproductive tract of pigs and is considered an opportunistic pathogen implicated in spontaneous abortion.

High-hygiene status increased the number of γ-proteobacteria on the ileal mucosa. All 16S rRNA gene libraries from the IR group contained members of the γ-proteobacteria class and grouped mainly with Enterobacteriaceae, including sequences identified as *E. coli *spp. with pathogenic properties which may pose a health risk for the young pig as well as the human population.

Members of the ε-proteobacteria were the second most abundant group within the Proteobacteria phylum and were represented by two major bacterial families, Helicobacteraceae and Campylobacteraceae. Most clones were obtained from the IN group and included bacteria of recognized pathogenic phenotype (13% of IN sequences).

### Transcriptomic analysis of gene expression patterns in the ileum of pigs from different environments

While the comprehensive profiling of the mucosa-adherent microbial community revealed large differences in composition attributable to differences in housing environment, a key goal of this study was to determine whether this translated into different host-specific gene responses. Therefore, an Affymetrix GeneChip microarray analysis was conducted on ileum tissue from the same site used for 16S rRNA gene library construction.

### Effects of treatment extremes on gene expression

Perhaps not surprisingly, mucosa-adherent microbial diversity in the ileum was most affected by experimental isolator housing, as this constituted a high-hygiene environment. To ascertain the differences in host-specific transcriptional responses between this treatment and the natural outdoor environment (*treatment extremes*), Affymetrix microarray analysis was performed on the comparison IR versus OUT at day 5 (neonatal stage), day 28 (weaning age) and day 56 (nearing maturity).

**Figure 6:**
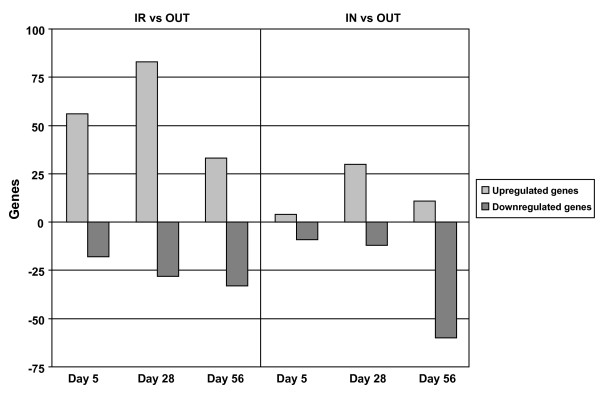
**Differentially expressed genes in the ileum of animals housed in different environments**. Differentially expressed genes at each time-point are shown for the two treatment comparisons (*P *< 0.01, -2 ≤ fold change ≥ 2, N = 6). Microbiota differences between the treatment groups were associated with large differences in gene expression in the ileum.

Seventy-four probesets were differentially expressed (*P *< 0.01 and -2 ≤ fold change ≥ 2) at the neonatal stage (Figure 6 and Additional file 2A). Fifty-six of these genes were highly expressed in the IR group, while 18 genes were higher in the OUT group. Interestingly, within the IR gene set, increased expression of genes that are closely linked to Type 1 interferon (IFN) signalling was observed. These genes included *IRF7*, *FAM14A*, *UBE2L6*, *GBP2 *and *USP18*. Some of the most highly-regulated genes (up to 11-fold higher in the IR group) were viperin, a tightly regulated *ISGF3 *target gene [22], and *IRP6*, a pig-specific gene homologous to human viperin. Another group showing increased expression in the IR group included 15 genes involved in cholesterol synthesis, such as *DHCR7*, *DHC24*, *SC5DL*, *HMGCS1*, *CYP51A1 *and *ERG1*. Genes of interest showing higher expression in the OUT group compared to the IR group included *TLR2 *as well as *HBB *and *HBA1*, both of which code for haemoglobin proteins.

At day 28, 111 genes were differentially expressed (Figure 6 and Additional file 2B). Twenty-one of the 83 transcripts expressed at higher levels in IR were the same as those found at day 5, and included the IFN-induced genes *IRF7 *and *GBP2*. Several other Type 1 IFN-induced genes (*G1P2*, *IFIT2*, *IFIT3*, *MX2*, *ISG20 *and *IFITM3*) were higher in IR animals compared to OUT animals, indicating a consistent treatment effect on Type 1 IFN signalling pathways. Also in common with the day 5 gene expression set, nine cholesterol synthesis genes were increased in the IR group. Consistent with these findings, microbiota-driven effects on cholesterol metabolism and trafficking have been previously documented [23]. Other transcripts expressed higher in the IR group compared to the OUT group included the chemokines *CXCL12*, *CCL28*, *CCL2*, *CCL8 *and *CXCL9*, the chemokine receptor *CCR5 *and the chemokine ligand *CCL4L*. *PMP22 *was increased in the OUT group. This gene is co-expressed with occludin and zona occludens 1 at tight junctions in epithelial cells [24].

Sixty-six genes were differentially expressed between IR and OUT at day 56 (Figure 6 and Additional file 2C). Some of the genes showing higher expression in the IR group included *KAI1*, *CEBPB*, *LTB4DH*, *COL14A1 *and *COL1A2*. Changes in *CEBPB *(CCAAT/enhancer-binding protein beta) expression in IR animals may be functionally important as this gene is involved in the regulation of inflammatory responses [25]. Notably, a group of T-cell-related genes was increased in OUT animals, including *TCA_HUMAN*, *LY96*, *CD8A*, *TRGV9*, *LCP1*, *LCP2*, *CXCL9 *and *TEC*, all of which are involved in T cell signalling, expansion, activation and trafficking. Other highly expressed transcripts in the OUT group included *EGR1*, *SELL *(important for leukocyte-endothelial cell interactions), *PIGR *(poly-Ig receptor) and *PIK3CG*.

Consistently, *PDK4 *was higher in the OUT group compared to the IR group at all three time-points. *PDK4 *has an important function in glucose metabolism, and its expression is regulated by glucocorticoids, retinoic acid and insulin; however, its potential relevance in host-microbe interactions is currently unknown.

**Figure 7:**
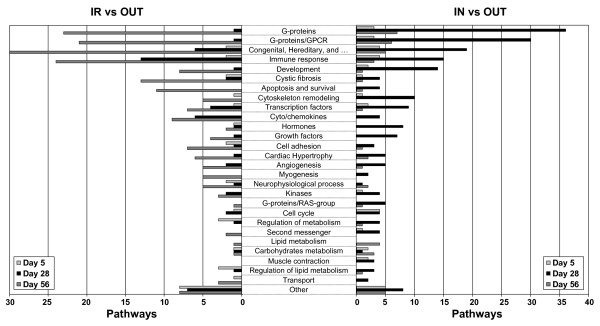
**MetaCore pathway analysis of differentially expressed genes of animals housed in different environments**. Differentially expressed genes (*P *< 0.05) were imported into GeneGo MetaCore analytical software to determine significantly enriched canonical pathways in each group. Data represent the distribution in cell process categories of statistically significantly enriched pathways (*P *< 0.05) of the comparisons IR vs OUT **(A) **and IN vs OUT **(B)**. Most pathways from both comparisons group into five categories: G-proteins; G-protein coupled receptor; congenital, hereditary and neonatal diseases and abnormalities; immune response; and development. Note that there is redundancy in category allocation.

Biological pathway analysis revealed that a large number of *Immune response *pathways were affected (Table 3 and Figure 7A). Other highly represented pathways included *G-protein *and *Congenital, hereditary and neonatal diseases and abnormalities*. Consistent with the analysis of individual gene data, the pathway for *Immune response-IFN alpha/beta signalling *was increased at day 28 and day 56 in the IR group compared to the OUT group. *Immune response-Antigen presentation by MHC class l *was affected at all three time-points and also higher in IR compared to OUT. Gene Ontology (GO)-enrichment analysis (Table 4) further confirmed these findings. While a number of GO categories were consistently affected by treatment, including *Immune response *(GO:0002376), the major biological process affected was *Antigen processing and presentation *(GO:0019882). Other affected GO-processes were *Antigen processing and presentation of peptide antigen via MHC class I *(GO:0002474), *Antigen processing and presentation of peptide antigen *(GO:0048002) and *Antigen processing and presentation of peptide or polysaccharide antigen via MHC class II *(GO:0002504).

**Table 3:** MetaCore pathway analysis of differentially expressed genes of the treatment comparison IR versus OUT.

**Day 5 IR vs OUT**	***P*-value**	Significant*	Total**
Cholesterol Biosynthesis	5.574E-13	15	21
Oxidative phosphorylation	0.00002	21	99
Vitamin B6 metabolism	0.00412	3	5
Cell adhesion_Gap junctions	0.00536	6	22
Regulation of lipid metabolism_Insulin regulation of fatty acid methabolism	0.00770	9	46
Regulation of lipid metabolism_Regulation of lipid metabolism via LXR, NF-Y and SREBP	0.00820	7	31
Cytoskeleton remodeling_Neurofilaments	0.01043	6	25
Ubiquinone metabolism	0.01169	9	49
Immune response_Antigen presentation by MHC class I	0.0127	6	26
Cell adhesion_Endothelial cell contacts by junctional mechanisms	0.0127	6	26
			
**Day 28 IR vs OUT**	***P*-value**	Significant*	Total**
Cholesterol Biosynthesis	0.00000	10	21
Immune response_Antigen presentation by MHC class I	0.00001	10	26
Immune response_Classic complement pathway	0.00003	12	40
Immune response_IFN alpha/beta signalling pathway	0.00005	9	24
Regulation of lipid metabolism_Regulation of lipid metabolism via LXR, NF-Y and SREBP	0.00209	8	31
Immune response_Antiviral actions of Interferons	0.00209	8	31
CFTR folding and maturation (norm and CF)	0.00320	5	14
Immune response_IL-22 signalling pathway	0.00321	8	33
Neurodisease_Parkin disorder under Parkinson's disease	0.00592	7	29
Immune response_Antigen presentation by MHC class II	0.00790	4	11
			
**Day 56 IR vs OUT**	***P*-value**	Significant*	Total**
Immune response_NFAT in immune response	0.00027	15	42
Immune response_Bacterial infections in normal airways	0.00040	14	39
Bacterial infections in CF airways	0.00048	15	44
Immune response_IL-12-induced IFN-gamma production	0.00066	12	32
Immune response_ICOS pathway in T-helper cell	0.00082	13	37
Immune response_CD28 signalling	0.00123	14	43
Cell adhesion_Integrin-mediated cell adhesion and migration	0.00201	14	45
Development_Angiopoietin - Tie2 signalling	0.00250	11	32
Apoptosis and survival_HTR1A signalling	0.00362	12	38
Sphingolipid metabolism/Human version	0.00362	12	38

Differentially expressed genes (*P *< 0.05) were imported into GeneGo MetaCore analytical software to determine the significantly enriched canonical pathways in the treatment comparison IR vs OUT. The top ten pathways for each comparison are shown, with the number of genes assigned to each pathway, and the corresponding *P*-value.

* The number of genes on each map that are differentially expressed in the specific treatment comparison

** The total number of genes on each map

**Table 4:** GO analysis of differentially expressed genes of animals housed in different environments.

		IR vs OUT						IN vs OUT					
GO Category	GO Term	Day 5		Day 28		Day 56		Day 5		Day 28		Day 56	
		Genes*	*P*-value	Genes*	*P*-value	Genes*	*P*-value	Genes*	*P*-value	Genes*	*P*-value	Genes*	*P*-value
GO:0019882	Antigen processing and presentation	62	2.4E-33	28	8.0E-06	26	5.1E-05	25	6.0E-10	26	1.8E-07	26	8.8E-06
GO:0002376	Immune system process	65	3.5E-21	42	4.3E-07	33	0.001	27	1.6E-06	30	5.4E-05	35	2.7E-05
GO:0002474	Antigen processing and presentation of peptide antigen via MHC class I	15	1.6E-08	16	8.6E-10	15	7.8E-09	14	3.0E-11	15	1.5E-10	15	2.2E-09
GO:0048002	Antigen processing and presentation of peptide antigen	15	1.6E-08	16	8.6E-10	15	7.8E-09	14	3.0E-11	15	1.5E-10	15	2.2E-09
GO:0006955	Immune response	54	1.8E-16	31	4.7E-04			16	0.021			24	0.015
GO:0002504	Antigen processing and presentation of peptide or polysaccharide antigen via MHC class II	36	1.5E-17										
GO:0050896	Response to stimulus	57	3.5E-07	41	0.018								
GO:0006412	Translation									13	0.002		
GO:0006807	Nitrogen compound metabolic process			9	0.007	8	0.021						
GO:0030163	Protein catabolic process			5	0.008								

Differentially expressed transcripts (*P *< 0.05) are shown assigned to the GO-category 'Biological Process'. The top ten GO-categories are shown. The number of transcripts for each function is shown, with the corresponding *P*-value.

*Genes involved in the specific GO-category

### Effects of housing environment on gene expression

Differences in ileal mucosa-adherent microbial composition between the IR group and the OUT group were associated with large host-specific transcriptional differences in the ileum. We next set out to assess whether the microbial differences associated with the IN and OUT environments had a similar impact on the gut transcriptome of the pig. While the number of differentially expressed genes between IN and OUT housed animals was smaller than between the treatment extremes (i.e. IR and OUT), similar trends could be discerned.

In the neonatal pig, the expression levels of 13 probesets were differentially expressed between the IN and OUT animals (Figure 6 and Additional file 2D). Nine genes were higher in IN animals, and this included *CXCL9*, which is involved in T cell trafficking. Four genes showed higher expression in OUT animals, including *TFRC*.

In weaning animals, 42 genes were differentially expressed between the two rearing environments (Figure 6 and Additional file 2E). Twelve transcripts were higher in IN animals, including *TAFA2 *(distantly related to *CCL3*), *CCR1 *and *CXCR4*. Of the 30 genes that were higher in the OUT group, genes of interest included *PMP22*, *CNKSR1*, *TJP4 *and *LTBR *(all increased between two- and three-fold).

The largest differences in gene expression were observed at day 56, when 71 genes were differentially expressed between the treatments (Figure 6 and Additional file 2F). Transcripts increased in IN animals (60 probesets in total) included three Type 1 IFN-inducible genes (*IFRD1*, *OAS1 *and *IFIT2*). The antibacterial peptide genes *LYZ *(Lysozyme C precursor), *PI3 *(elafin precursor) and *BPI *(bactericidal permeability-increasing protein precursor) were increased 6.92-, 6.8- and 2.93-fold, respectively, in IN animals compared to OUT animals and may contribute to the observed differences in microbiota composition between these groups. Furthermore, these peptides appear to maintain gut homeostasis as evidenced by their aberrant expression in Crohn's Disease [26] and Ulcerative Colitis [27]. *CCL*8 (a monocyte chemotactic protein) was also higher in the IN group. Some of the 11 genes increased in OUT animals were *PMP22 *and *SELL*, in accordance with the observations from the IR and OUT comparison.

The most affected pathways belonged to *Immune response*, *G-protein *and *Congenital, hereditary, and neonatal diseases and abnormalities *(Table 5 and Figure 7B), as observed previously in the treatment extremes comparison.

**Table 5:** MetaCore pathway analysis of differentially expressed genes of the treatment comparison IN versus OUT.

Day 5 IN vs OUT	*P*-value	Significant*	Total**
Transcription_Ligand-Dependent Transcription of Retinoid-Target genes	0.00022	7	32
G-protein signalling_G-Protein alpha-s signalling cascades	0.00473	5	28
Transcription_CREM signalling in testis	0.00626	4	19
Proteolysis_Putative ubiquitin pathway	0.01076	4	22
Immune response_PGE2 signalling in immune response	0.01102	5	34
Development_Lipoxin inhibitory action on PDGF, EGF and LTD4 signalling	0.01468	4	24
Inhibitory action of Lipoxin A4 on PDGF, EGF and LTD4 signalling	0.01468	4	24
Cell cycle_Initiation of mitosis	0.01694	4	25
Cell cycle_Regulation of G1/S transition (part 1)	0.01748	5	38
Muscle contraction_GPCRs in the regulation of smooth muscle tone	0.02082	6	54
			
**Day 28 IN vs OUT**	***P*-value**	Significant*	Total**
Cytoskeleton remodeling_CDC42 in cellular processes	0.00013	7	22
Oxidative stress_Role of ASK1 under oxidative stress	0.00013	7	22
Immune response_Histamine H1 receptor signalling in immune response	0.00028	9	40
Development_VEGF signalling and activation	0.00044	8	34
Cytoskeleton remodeling_TGF, WNT and cytoskeletal remodeling	0.00079	15	107
Cytoskeleton remodeling_Cytoskeleton remodeling	0.00079	14	96
Immune response_IL-3 activation and signalling pathway	0.00108	7	30
Immune response_Histamine signalling in dendritic cells	0.00118	8	39
Development_TGF-beta receptor signalling	0.00135	9	49
Signal transduction_Activation of PKC via G-Protein coupled receptor	0.00267	8	44
			
**Day 56 IN vs OUT**	***P*-value**	Significant*	Total**
Peroxisomal branched chain fatty acid oxidation	0.00004	8	22
Cholesterol Biosynthesis	0.00024	7	21
Neurophysiological process_Dopamine D2 receptor transactivation of PDGFR in CNS	0.00068	6	18
Propionate metabolism p.1	0.00196	5	15
Development_Angiotensin signalling via beta-Arrestin	0.00281	6	23
G-protein signalling_G-Protein alpha-12 signalling pathway	0.00448	7	33
Delta508-CFTR traffic/ER-to-Golgi in CF	0.00783	4	13
G-protein signalling_Rap2B regulation pathway	0.00783	3	7
Development_Mu-type opioid receptor signalling via Beta-arrestin	0.00962	5	21
Mitochondrial unsaturated fatty acid beta-oxidation	0.01709	4	16

Differentially expressed genes (*P *< 0.05) were imported into GeneGo MetaCore analytical software to determine the significantly enriched canonical pathways in the treatment comparison IN vs OUT. The top ten pathways for each comparison are shown, with the number of genes assigned to each pathway, and the corresponding *P*-value.

* The number of genes on each map that are differentially expressed in the specific treatment comparison

** The total number of genes on each map

### Real-time quantitative PCR to analyze differentially expressed genes

Real-time PCR was performed for *CCL28*, *CCL8*, *CXCL12*, *CXCL9*, *CXCR4*, *IFIT2*, *FKBP5*, *IRF7*, *IRP6*, *MT1J*, *MX*, *PDK4*, *PI3, SELL*, *SQLE *and *TFRC*. These genes were selected from the gene expression data set both because they showed significant changes and because of their involvement in key immune system pathways. Verification of the true differential expression between treatment groups of these genes by Real-time PCR was therefore considered essential for further biological interpretation.

**Figure 8:**
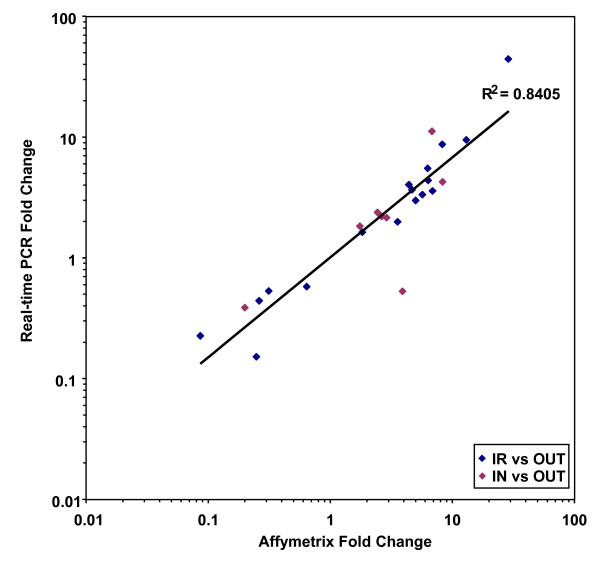
**Scatterplot of concurrence between Affymetrix microarray data and Real-time PCR data**. Correlation between mean fold change values of both comparisons obtained by Affymetrix microarray analysis and Real-time PCR analysis. The diagonal line represents the power trendline (R^2 ^= 0.8405).

The subsequent correlation between Affymetrix microarray and Real-time PCR data (R^2 ^= 0.8405; *P *< 0.001; Figure 8) was positive, further substantiating the biological importance of the selected genes and identified pathways. Real-time PCR verification for the comparison IR versus OUT showed that direction and magnitude of fold change correlated well with the Affymetrix microarray results (Table 6). In some cases the fold changes detected by Real-time PCR were lower than those observed by microarray analysis.

**Table 6:** Verification of microarray results by Real-time PCR.

		Real time PCR IR vs OUT					Affymetrix microarray	
Gene	Day	IR*	OUT*	SED**	Fold Change	*P*-value	Fold Change	*P*-value
*FKBP5*	5	5.56 ± 0.76	4.77 ± 0.72	0.42	-1.73	0.092	-1.56	0.014
*IRF7*	5	6.12 ± 1.43	7.70 ± 1.04	0.72	2.99	0.056	5.00	0.003
*IRP6*	5	5.62 ± 2.97	8.75 ± 1.54	1.37	8.71	0.054	8.28	0.008
*MT1J*	5	2.31 ± 3.61	7.78 ± 1.88	1.66	44.33	0.012	28.70	0.002
*MX*	5	6.20 ± 2.35	8.08 ± 0.45	0.98	3.66	0.110	4.67	0.017
*PDK4*	5	9.30 ± 1.13	8.39 ± 0.38	0.49	-1.88	0.111	-3.19	0.005
*SQLE*	5	5.85 ± 1.51	7.59 ± 1.44	0.85	3.34	0.069	5.69	0.044
*CCL8*	28	8.28 ± 0.87	10.29 ± 1.29	0.68	4.04	0.021	4.41	0.000
*CXCL12*	28	4.44 ± 0.23	5.15 ± 0.27	0.14	1.63	0.001	1.83	0.003
*CXCL9*	28	4.91 ± 1.30	7.37 ± 2.36	1.10	5.52	0.057	6.28	0.007
*IRF7*	28	5.21 ± 1.55	6.20 ± 0.70	0.69	1.99	0.196	3.56	0.006
*MT1J*	28	3.33 ± 2.10	6.58 ± 1.58	1.07	9.49	0.014	13.01	0.001
*PDK4*	28	9.75 ± 0.66	7.61 ± 0.78	0.42	-4.43	0.000	-11.62	0.000
*SQLE*	28	5.14 ± 1.12	7.27 ± 2.04	0.95	4.38	0.056	6.34	0.001
*TFRC*	28	5.78 ± 1.38	7.62 ± 0.89	0.67	3.58	0.024	6.88	0.000
*PDK4*	56	8.73 ± 0.54	7.55 ± 0.42	0.28	-2.27	0.002	-3.82	0.007
*SELL*	56	18.82 ± 0.88	16.10 ± 3.29	1.39	-6.60	0.101	-4.03	0.003
		**Real time PCR IN vs OUT**					**Affymetrix microarray**	
**Gene**	**Day**	IN*	OUT*	SED**	**Fold Change**	***P*-value**	**Fold Change**	***P*-value**
*TFRC*	5	9.15 ± 0.64	7.78 ± 1.20	0.56	-2.58	0.041	-5.01	0.005
*CXCR4*	28	9.09 ± 1.01	8.17 ± 1.18	0.63	-1.89	0.177	3.91	0.010
*CCL28*	56	8.61 ± 0.74	9.75 ± 0.95	0.49	2.20	0.044	2.64	0.025
*CCL8*	56	9.19 ± 0.80	10.44 ± 1.31	0.63	2.38	0.080	2.44	0.009
*PI3*	56	5.67 ± 1.26	9.16 ± 1.28	0.82	11.17	0.004	6.80	0.002
*IFIT2*	56	5.63 ± 2.12	7.72 ± 1.79	1.13	4.26	0.095	8.32	0.009
*PDK4*	56	6.68 ± 0.25	7.55 ± 0.42	0.20	1.83	0.002	1.75	0.008
*SQLE*	56	6.30 ± 0.92	7.40 ± 0.93	0.53	2.15	0.065	2.90	0.011

Real-time PCR results at each time-point for the comparisons IR vs OUT and IN vs OUT.

*PCR values are expressed as mean ΔCt ± SD, N = 6.

**Standard error of difference

The differentially expressed genes from the IN versus OUT comparison were examined by Real-time PCR and again correlated well with the Affymetrix microarray results (Table 6). Only *CXCR4 *expression in the IN group showed disagreement between the two platforms, as it was increased using microarray analysis, and decreased using Real-time PCR.

## Discussion

The current study sought to investigate the effects of environmental hygiene on microbial colonization and composition of the gut microbiota. Additionally, transcriptomic profiling was performed to assess the impact of environmental hygiene on gene expression, in particular those genes and pathways associated with immune function. Both indoor and isolator (representing urban lifestyle and high-hygiene status, respectively), and outdoor (representing rural lifestyle and low-hygiene status) conditions were compared using pigs as an experimental model.

Using extensive analysis of 16S rRNA gene libraries our study categorically revealed that early-life environment has a major impact on microbial diversity and that these differences are sustainable throughout adult life. Many of the bacterial phylotypes identified in our study are commonly found in the human and animal gastrointestinal tract [28-30]. Our results also identified that only 3.3% of the clones had less than 97% sequence similarity to existing database entries.

A major finding of the current study was the significant increase in the Firmicutes phylum in sow-reared pigs housed in outdoor environments compared to littermates housed in isolators with daily antibiotic treatment. Within the Firmicutes phylum, the most compelling observation was the abundance of lactobacilli in animals reared in the outdoor environment. Lactobacilli are often associated with the suckling pig and early stages of colonization in the gastrointestinal tract. In this study, the high abundance of lactobacilli in the fecal samples obtained from truly adult sows identified lactobacilli as normal colonizers of the adult pig microbiota in the outdoor environment. Leser *et al*. [28] found similar high-abundance phylotypes associated with the ileum, including *L. amylovorous*, *L. johnsonii *and *L. reuteri*, in pigs from different rearing environments. Our study further revealed that an increase in hygiene status in pigs housed both indoor and in isolators with antibiotic administration was associated with a significant decrease in mucosa-adherent lactobacilli. Affected species included *L. reuteri*, *L. delbrueckii*, *L. amylovorous*, *L. johnsonii *and *L. mucosae*.

The reduced microbial diversity in outdoor animals compared to indoor and isolator housed groups was a somewhat surprising outcome. These outdoor animals were exposed to a huge variety of different bacterial species, as well as fungi, Archaea and viruses, originating from both maternal and environmental sources. The soil especially is hugely abundant in micro-organisms, and estimates of soil diversity show the presence of at least 32 phyla, the dominant members of which are Proteobacteria, Bacteroidetes and Firmicutes [31]. Soil ecosystems potentially provide an important source of microbes for gut colonization of outdoor animals. However, only a selective subset of environmental bacteria colonize the intestine, since we noted that the pig gut microbiota was comprised of a restricted number of phyla, dominated by Bacteroidetes and Firmicutes, consistent with published findings on the diversity of the adult human gut [29]. Current thinking has focussed on the benefits of a highly diverse gut microbiota, as it has long been considered that this confers greater plasticity of the bacterial community to respond to perturbations within the gut ecosystem [17]. Paradoxically, we found that exposure to a large variety of environmental microbes in early life does not generate greater diversity in the adult gut but rather leads to a microbiota that is dominated by a limited number of phyla composed of bacteria with proven health-promoting properties.

Lactobacilli have long been known for their health-promoting effects and they directly limit the prevalence of several intestinal pathogens including *E. coli *and salmonella [32-34]. In this study, *L. reuteri *was one of the most abundant members of the mucosa-adherent microbiota of the outdoor group. Reuterin, a broad-spectrum antimicrobial substance, is produced by *L. reuteri *[35] and inhibits most intestinal bacteria with the exception of *Lactobacillus *strains [36]. Importantly, the greater abundance of *L. reuteri *in the outdoor animals may contribute to the enhanced presence of other *Lactobacillus *species as well as the decreased microbial diversity observed in these animals. A further point meriting comment is the reduced presence of potentially pathogenic phylotypes in outdoor-housed pigs. These phylotypes were clearly present in both indoor and isolator housed animals, although animals showed no overt signs of infection. The specific reduction in Firmicutes, in particular lactobacilli, in these pigs may affect the normal mechanisms of colonization resistance that control potentially pathogenic populations within the gut ecosystem.

Although there has been a major focus on health-promoting probiotic actions of lactobacilli following their introduction as oral supplements, significantly less attention has been paid to the effects of naturally-acquired, gut-colonizing (autochthonous) lactobacilli. Given that immune modulation is dependent on gut colonization, close proximity to the mucosa and host adaptation, naturally-acquired lactobacilli clearly deserve greater attention. Of those species studied, *L. casei*, *L. johnsonii *and *L. plantarum *are strong inducers of IL-12 and/or INF-γ, thereby favouring a Th1 cytokine profile [37,38]. Conversely, *L. reuteri *inhibits the induction of IL-12 and TNF-α and also attenuates *L. casei*-induced IL-12 [38]. A fine balance between Th1-polarising lactobacilli strains and those which counterbalance such responses may be an important factor in maintaining mucosal immune homeostasis and explain the lack of overt Th1 or Th2 responses in outdoor-housed pigs in the current study.

While there was no evidence of Th1/Th2 pathways being affected, we found significant effects of environment on the Type 1 interferon (IFN) signalling pathways. Isolator-reared pigs exhibited increased gene expression levels of the IFNα/β transcription/signalling factors *IRF7 *and *USP18*. Type 1 IFN signalling induces the expression of a large number of target genes, which in the current study included *MX2*, *G1P2*, *ISG20*, *FAM14A, IFIT2 *and *IFIT3*. Three Type 1 IFN-inducible genes (*IFRD1*, *OAS1 *and *IFIT2*) were increased in indoor-housed animals compared to outdoor-housed animals, indicating that the IFNα/β pathway is directly affected by the housing environment. A number of recent studies further support our data describing the influence of the gut microbiota on the Type 1 IFN pathway. For example, conventionalized pigs exhibited increased expression of *IRF7*, *STAT1 *and *STAT2 *when compared with their germ-free counterparts [39]. Conversely, bacterial colonization of germ-free mice led to a decreased expression of the IFN-related genes *IRF7*, *ISGF3G*, *IFIT1* and *STAT1*[40]. Our study further qualifies these findings by establishing that specific microbial composition, rather than the microbiota as such, influences Type 1 IFN signalling during early colonization and development.

Type 1 IFNs have many biological properties, including innate, cellular and humoral adaptive immune responses [41]. Much evidence has focussed on their central role in pathogen resistance, especially viral immunity through recognition of dsRNA. The significance of Type 1 IFNs in response to bacterial colonization and infection is receiving increasingly more attention [42,43]. IFN expression is induced in numerous cell lineages, including macrophages and plasmacytoid dendritic cells, by bacterial components such as LPS and CpG DNA [44-46]. It is worth noting that the transcriptome analysis was performed on whole ileal tissue samples, rather than on a specific cell subset. In this study we elected to study interactions and contributions of all cell lineages present in the gut to comprehensively characterize the transcriptomic changes induced by different microbiota compositions. However, the contribution of individual lineages such as plasmacytoid dendritic cells (DCs), which naturally produce Type 1 IFN, will be addressed in subsequent studies.

IFN-α/β has profound effects on immune cell development [41] by regulating the differentiation of B and T cells, myeloid DCs and natural killer cells. Activation of immature DCs by IFN-α/β upregulates major histocompatibility complex (MHC) class I. Consistent with this, we found that antigen presentation by MHC class I was also affected by the microbiota and was upregulated in indoor reared animals which also displayed increased Type 1 IFN levels. MHC class I molecules are Type 1 IFN-inducible genes whose promoter regions contain typical IFN-stimulated response elements (ISREs). MHC class I molecules are specialized for presentation of endogenously synthesized proteins, including self-proteins, to the TCR of CD8+ T-cells [47]. The cross-presentation of antigens on MHC class I molecules, the induction of CTL responses and the subsequent memory CD8+ T cell survival are also dependent on IFN-α/β.

Increased expression of MHC class I in the indoor environment was accompanied by the upregulation of a plethora of chemokines, including *CCL2*, *CCL8*, *CCL28*, *CCR1*, *CXCR4 *and *CXCL12*. Chemokines are chemotactic cytokines that function during immune responses to recruit effector cells to sites of inflammation and infection. They are involved in the pathophysiology of many diseases. Numerous chemokines have been implicated in the pathology and perpetuation of tissue destructive inflammatory processes in patients with IBD, including *CCL2 *[48] and *CCL8 *[49]. Increased expression of these chemokines in the indoor-housed animals indicates the presence of an immune-activated gut microenvironment. This contrasts with the lack of innate and pro-inflammatory gene expression in the outdoor-housed animals, which may be indicative of a more immune-tolerant and homeostatic mucosal immune system in these animals. Further studies are required to assess the impact of the microbiota, immune gene transcription and immune cell lineages on specific tolerance towards food and environmental antigens and long-term predisposition to infection, food intolerance and allergy.

## Conclusion

Environmental exposure in early life has a significant impact on microbiota composition of the adult gut and the immune transcriptome during development. Rural, outdoor environments support the establishment of a natural microbiota dominated by lactobacilli and containing low numbers of potentially pathogenic bacteria and this may be an important factor in maintaining mucosal immune homeostasis and limiting excessive inflammatory responses in the gut. The significance of the microbiome and transcriptome data presented herein in relation to immune events such as oral tolerance and host defence against enteric pathogens is a major focus of our future studies.

## Methods

### Experimental animals and tissue collection

Twelve Large White × Landrace sows (*Sus scrofa*) were housed at either an indoor (intensive) or an outdoor (extensive) facility. The sows were artificially inseminated by the same boar to minimize genetic variation among the offspring. Three piglets from each outdoor-housed sow (OUT) and indoor-housed sow (IN) were left to suckle with the mother until day 28, when all piglets were weaned. Three piglets from each indoor-housed sow (18 piglets in total) were transferred to individual isolator units at the School of Clinical Veterinary Science (University of Bristol, UK) at 24 hours of age. These piglets were given a daily dose of antibiotic cocktail (Baytril (Bayer Healthcare, Uxbridge, UK) and Amoxinsol 50 (Vétoquinol UK Ltd., Buckingham, UK)) for the duration of the study. Up until day 28, the isolator-housed piglets (IR) were fed commercial porcine milk replacer (PiggiMilk, Parnutt Foods Ltd., Sleaford, UK) dispensed by an automated liquid feeding system. From day 29 onwards, all piglets were fed creep feed (Multiwean, SCA Nutrition Ltd, Thirsk, UK) *ad libitum*. The experiment was run in three consecutive replicates, using four sows and 18 piglets in every replicate.

Six randomly chosen piglets per treatment group were sacrificed by injection of sodium pentobarbitone (Euthesate, Willows Francis Veterinary Ltd, Crawley, UK) at time-points on day 5, 28 and 56. The ileum, defined as the region corresponding to 75% in length from the pyloric sphincter, was excised. Detailed molecular analysis was performed on this site as it represents a key region involved in both immune-inductive and effector activities, including bacterial antigen sampling. Two ileal tissue samples were taken and either washed in ice-cold phosphate buffered saline (PBS)/0.1% Tween 20 (Sigma-Aldrich, Gillingham, UK) for construction of mucosa-associated 16S rRNA gene libraries (10-cm piece) or processed in ice-cold PBS and transferred to RNAlater (Applied Biosystems, Warrington, UK) for Affymetrix microarray and Real-time PCR studies (2-cm piece). All animal work was performed according to the institutional and Home Office UK ethical guidelines.

### Analysis of the mucosal microbiota

Gut contents (N = 4 per treatment group) were removed from the ileum, and the tissue was washed with ice-cold PBS and incubated in ice-cold PBS/0.1% Tween 20 overnight. Detached bacteria were harvested by centrifugation at 10,000 × g for 10 min at 4°C. Total DNA from the pellet was isolated using a DNA Spin Kit for Soil^® ^(QBiogene Inc., Cambridge, UK) according to the manufacturer's protocol. PCR amplification of the 16S rRNA genes was carried out with the universal primer set S-D-Bact-0008-a-S-20 (5'-AGAGTTTGATCMTGGCTCAG-3'; positions 8 to 27 in the *Escherichia coli *16S rRNA gene) and S-*-Univ-1492-a-A-19 (5'-ACGGCTACCTTGTTACGACTT-3'; positions 1510 to 1492) [50]. Primer positions are represented according to the OPD nomenclature [51]. PCR cycling conditions were one cycle at 94°C for 5 min, followed by 25 cycles at 94°C for 30 sec, 57°C for 30 sec, 72°C for 2 min, with a final extension at 72°C for 10 min. PCR products were purified with the Wizard^® ^SV Gel & PCR Clean-up System (Promega, Southampton, UK), cloned into the pCR-4 cloning vector and transformed into *E. coli *TOP 10 chemically competent cells (TOPO TA Cloning Kit; Invitrogen, Paisley, UK) according to the manufacturer's instructions. Recombinant colonies were picked and the inserts were sequenced in the RINH genomics facility (University of Aberdeen, UK) using the primer set S-*-Univ-0907-a-A-20 (5'CCGTCAATTCATTTGAGTTT-3') and S-*-Univ-0519-a-A-18 (5'-GWATTACCGCGGCKGCTG-3') [50]. All clone libraries were constructed under identical conditions in order to minimize sample-to-sample variation, thus the relative differences in microbial composition between the samples truly reflect animal treatment differences.

### Enumeration of *Lactobacillus *species

Approximately 70 mg gut contents from IR and OUT animals at day 56 from both the ileum (IR: N = 3; OUT: N = 2) and the colon (IR: N = 4; OUT: N = 3) were transferred to Hungate tubes containing 2 ml of MRS broth/0.2% Tween 80 (Oxoid, Basingstoke, UK) and dispersed by vortexing. The gut content suspensions were diluted in a series of seven sequential ten-fold dilutions. Twenty microlitre aliquots of the dilutions were plated out on MRS agar plates and dried off. The plates were placed in an anaerobic gas jar and incubated at 37°C. Plates were read and data recorded and calculated after 48 hours of incubation.

### Sequence alignment and phylogenetic analysis

Sequences were assembled using Lasergene 6 software (DNASTAR Inc.; Infogen Bioinformatics, Broxburn, UK) and tested for possible chimeras using Chimera Check v2.7 (online analysis at RDP-II website, http://rdp8.cme.msu.edu/cgis/chimera.cgi?su=SSU and Bellerophon [52]http://foo.maths.uq.edu.au/~huber/bellerophon.pl. Sequences with no close neighbours in RDP-II were additionally subjected to Basic Local Alignment Search Tool (BLAST) analysis http://www.ncbi.nlm.nih.gov/BLAST. Chimeric and poor quality sequences were excluded from further phylogenetic analysis.

The resulting 16S rRNA gene contigs were aligned using Multiple Sequence Comparison by Log-Expectation (MUSCLE, http://www.ebi.ac.uk/Tools/muscle[53]) and the alignments were inspected manually. The distance matrix (generated from the multiple sequence alignment) was calculated using the Dnadist application of the Phylogeny Inference Package http://evolution.genetics.washington.edu/phylip.html and Jukes-Cantor distance of 0.01. This stringent phylotype definition at 99% cut-off was used in part because evidence suggests that bacteria with nearly-identical 16S rRNA sequences may represent variable genotypes and different species [29].

Rarefaction and collector's curves of observed phylotypes, richness estimates and diversity indices were determined with the DOTUR program [54] using Jukes-Cantor corrected distance matrix. The bias-corrected Chao 1 richness estimator was calculated after 1000 randomizations of sampling without replacement. Collector's curves of observed and estimated (Chao 1 and the abundance-based coverage estimator, ACE) richness were constructed. Diversity was estimated using the Shannon (H) and Simpson indices (D). The Simpson reciprocal index was calculated as 1/D, and another version of the Simpson diversity index as 1-D. The Good's coverage percentage was calculated with the formula [1-(n/N)] × 100, where n is the number of phylotypes in a sample represented by one clone (singletons) and N is the total number of sequences in that sample [55].

Similarity search of the 16S rRNA gene sequences against database entries was performed using the BLAST program at the National Center for Biotechnology Information (NCBI) website http://www.ncbi.nlm.nih.gov/BLAST. By using a >99% sequence similarity criterion, the sequences were assigned to the respective bacterial phylotypes.

Phylotype comparisons were made among groups of subjects using the Mann-Whitney U test. Multiple comparisons were carried out using the Kruskal-Wallis test, with *P *< 0.05 considered statistically significant.

### Microarray hybridizations and data analysis

Ileal tissue (200 mg) (N = 6 per treatment group/time-point) was removed from RNAlater and lyzed in Trizol (Invitrogen). RNA was isolated using standard chloroform/isopropanol steps. Total RNA was further extracted with the RNeasy kit (Qiagen, Crawley, UK) according to the manufacturer's instructions, including an RNase-free DNase I (Qiagen) digestion step. RNA integrity was determined using the Agilent 2100 Bioanalyzer (Agilent Technologies, Wokingham, UK).

Eight microgram of total RNA was reverse transcribed to cDNA and then transcribed into biotin-labelled cRNA using the One-Cycle Target Labeling Kit (Affymetrix, Santa Clara, CA) according to the manufacturer's instructions. cRNA quality was determined by Agilent 2100 Bioanalyzer. Hybridization to the GeneChip Porcine Genome Array (Affymetrix) on a GeneChip Fluidics Station 450 (Affymetrix) was performed at the Institute of Medical Sciences Microarray Core Facility (University of Aberdeen, UK). Chips were scanned with an Affymetrix GeneChip Scanner 3000 (Affymetrix). Image quality analysis was performed using Gene Chip Operating Software (GCOS) (Affymetrix).

Further quality analysis, normalization by GeneChip Robust Multiarray Averaging (gcRMA), statistical analysis and heatmap generation was performed with the freely available software packages R http://www.r-project.org and Bioconductor http://www.bioconductor.org[56]. In particular we used the moderated F-test provided by the Bioconductor package *limma *to test for differential expression [57].

Statistical analysis was performed separately for each of the three time-points (day 5, 28 and 56) on the two group comparisons IR vs OUT and IN vs OUT. As detailed in the first Methods subsection, the animal experiments consisted of three replicates with two piglets in each of the three experimental groups. This has created a three-group design, with six biologically independent samples in each group and replicate as an additional blocking factor.

To address the multiple testing issue the Storey method [58] was used to calculate q-values, as implemented in the Bioconductor package *qvalue*. This method gives estimates of the associated false discovery rate for a given cut-off. Although these q-values are shown in Additional file 2, the lists of differentially expressed genes were not based only on q-values or *P*-values, but tried to address the balance between statistical significance and biological relevance. Thus, differences in gene expression between treatments were determined using a cut-off of *P *< 0.01 and -2 ≤ fold change ≥ 2. This approach is very much in line with recommendations based on the Micorarray Quality Control study (MAQC) [59], which recommends the use of fold change ranking plus a non-stringent *P *cutoff as a baseline practice in order to generate more reproducible differentially expressed gene lists.

Microarray data were submitted to the NCBI Gene Expression Omnibus (accession number GSE15256; http://www.ncbi.nlm.nih.gov/geo).

### Functional analysis of microarray data

Gene Ontology (GO) based functional interpretation of the data was performed using the Database for Annotation, Visualization and Integrated Discovery (DAVID 2006; http://david.abcc.ncifcrf.gov), an expanded version of the original web-accessible programs described by Dennis *et al*. [60]. Significantly different transcripts (*P *< 0.05) were allocated into the GO category *Biological Process *to unearth patterns of gene expression significantly enriched for specific GO terms.

All differentially expressed genes (*P *< 0.05) were imported into MetaCore analytical software (GeneGo, St Joseph, MI) to generate pathway maps. MetaCore is a proprietary, manually curated database containing human protein-protein, protein-DNA and protein compound interactions, metabolic and signalling pathways, and the effects of bioactive molecules. MetaCore software contains approximately 450 canonical signalling and metabolic pathways. Porcine Affymetrix probeset IDs were converted into human Affymetrix probeset IDs using annotation supplied by Tsai *et al*. [61]. Integrated pathway enrichment analysis was performed using the knowledge-based canonical pathways and endogenous metabolic pathways. Ranking of relevant integrated pathways was based on *P*-values calculated using hypergeometric distribution. *P*-values represented the probability of a given number of genes from the input list to match a certain number of genes in the map by chance, considering the numbers of genes in the experiment versus the number of genes in the map within the full set of all genes on maps.

### Real-time PCR analysis of differentially expressed genes

The mRNA levels differentially expressed between the treatment groups in microarray analyses were further validated using Real-time PCR. Two micrograms of total RNA isolated from the ileum (N = 6, isolated for microarray analysis) was reverse transcribed into cDNA using the High Capacity cDNA Reverse Transcription Kit (Applied Biosystems) with random primers. Real-time PCR analysis was performed using a 7500 Fast Real-Time PCR System (Applied Biosystems) with the Power SYBR Green PCR Master Mix (Applied Biosystems) according to the manufacturer's recommendations. Primers (Sigma-Aldrich; Additional file 3) were designed for the porcine sequence of interest using Primer Express Software v3.0 (Applied Biosystems). PCR cycling conditions were one cycle at 95°C for 10 min, followed by 40 cycles at 95°C for 15 sec and 60°C for 1 min, ending with a dissociation step. All samples were run in triplicate. *EEF1A1 *was selected as a reference gene for normalization due to its low variation between samples in the microarray analysis.

Data were analyzed on a logarithmic scale with base 2 by Student's t-test allowing for unequal variances with *P *< 0.05 considered statistically significant. Standard errors of differences were also calculated on this scale. Differences were back-transformed to calculate fold changes.

## Abbreviations

ACE: abundance-based coverage estimator; DCs: dendritic cells; GO: Gene Ontology; IFN: interferon; IN: indoor-housed piglets reared on the sow; INS: fecal samples from adult sows from indoor environment; IR: isolator-housed piglets receiving daily doses of antibiotics; MHC: major histocompatibility complex; MRS: De Man, Rogosa and Sharpe; OTU: operational taxonomic unit; OUT: outdoor-housed piglets reared on the sow; OUTS: fecal samples from adult sows from outdoor environment; PBS: phosphate buffered saline; PCR: Polymerase Chain Reaction; RDP: Ribosomal Database Project.

## Authors' contributions

DK, CRS, MB, JRP and BPG conceived and coordinated the study. IEM carried out the microarray experiment, bioinformatics analysis and RT-PCR work and drafted the manuscript along with BS and DK. BS carried out the 16S rRNA gene library preparation, constructed sequence contigs and performed phylogenetic identification. CRS, ML and MB conducted the animal trial. CDM helped with the statistical design of the study and developed the analysis strategy for the microarray and Real-time PCR data. CCM carried out the phylogenetic analysis of clone libraries. RIA and JIP were involved in technical and scientific discussion of the project. All authors read and approved the final manuscript.

## Supplementary Material

Additional file 1 Enumeration of *Lactobacillus *species. Click here for file

Additional file 2 **Transcripts differentially expressed between treatments at all three time-points.** Differentially expressed genes at each time point are shown for the comparison IR versus OUT, and IN versus OUT (*P *< 0.01, -2 ≤ fold change ≥ 2, N = 6). Click here for file

Additional file 3 **Real-time PCR primer sequences.** Porcine gene-specific primers were designed for the sequence of interest using Primer Express Software v3.0 (Applied Biosystems). Click here for file
