# Supplementary material for: Environmentally-acquired bacteria influence microbial diversity and natural innate immune responses at gut surfaces
Source: BMC Biol. 2009 Nov 20;7:79. doi: 10.1186/1741-7007-7-79 (PMC2785767; doi:10.1186/1741-7007-7-79)
Supplement: Additional file 1 — Enumeration of Lactobacillus species. [file 1741-7007-7-79-S1.DOC]

**Additional file 1 – Enumeration of *Lactobacillus* species**

| **Tissue site** | **Treatment** | **Mean (log_10_ CFU/g)** | **Range (log_10_ CFU/g)** |
| --- | --- | --- | --- |
| Ileum (N=3) | IR | 2.26 | 0-3.48 |
| Ileum (N=2) | OUT | 6.29 | 5.16-7.42 |
| Colon (N=4) | IR | 4.59 | 3.65-5.12 |
| Colon (N=3) | OUT | 7.25 | 5.30-8.32 |
