# Supplementary material for: Environmentally-acquired bacteria influence microbial diversity and natural innate immune responses at gut surfaces
Source: BMC Biol. 2009 Nov 20;7:79. doi: 10.1186/1741-7007-7-79 (PMC2785767; doi:10.1186/1741-7007-7-79)
Supplement: Additional file 2 — Transcripts differentially expressed between treatments at all three time-points. Differentially expressed genes at each time point are shown for the comparison IR versus OUT, and IN versus OUT (P < 0.01, -2 ≤ fold change ≥ 2, N = 6). [file 1741-7007-7-79-S2.DOC]

**Additional file 2A**

| **Day 5 IR vs OUT** | | | | | |
| --- | --- | --- | --- | --- | --- |
| **affy.id** | **FC** | **P-value** | **Q-value** | **Gene Name** | **Product** |
| Ssc.26063.1.S1_at | 10.69 | 0.0000 | 0.0982 | INSIG1 | Insulin-induced protein 1 |
| Ssc.15598.1.S1_at | 3.78 | 0.0000 | 0.1259 | INSIG1 | Insulin-induced protein 1 |
| Ssc.19659.2.S1_at | 3.52 | 0.0000 | 0.1259 | NP_005759 | Gene rich cluster, C3f gene [Homo sapiens] |
| Ssc.19659.1.S1_at | 2.62 | 0.0000 | 0.1736 | NP_005759 | Gene rich cluster, C3f gene [Homo sapiens] |
| Ssc.3253.1.S1_at | 2.25 | 0.0000 | 0.1736 | NSDHL | NAD(P)-dependent steroid dehydrogenase (H105e3 protein) |
| Ssc.5045.1.S1_at | 7.14 | 0.0001 | 0.1736 | EBP | 3-beta-hydroxysteroid-delta(8),delta(7)-isomerase (Cholestenol delta-isomerase) (Delta8-delta7 sterol isomerase) (D8-D7 sterol isomerase) (Emopamil-binding protein) |
| Ssc.12456.1.A1_at | 5.26 | 0.0001 | 0.1736 | NP_115821 | MEGF11 protein [Homo sapiens] |
| Ssc.16159.1.S1_at | 17.65 | 0.0001 | 0.1936 | SCD | Acyl-CoA desaturase (Stearoyl-CoA desaturase) (Fatty acid desaturase) (Delta(9)-desaturase) |
| Ssc.26820.1.A1_at | -2.94 | 0.0002 | 0.3904 | SLC39A10 | Solute carrier family 39 (zinc transporter), member 10 |
| Ssc.17934.1.S1_at | 6.53 | 0.0004 | 0.6512 | STARD10 | PCTP-like protein (PCTP-L) (StAR-related lipid transfer protein 10) (StARD10) (START domain-containing protein 10) (CGI-52) (Serologically defined colon cancer antigen 28) (Antigen NY-CO-28) |
| Ssc.30869.1.S1_at | 2.78 | 0.0004 | 0.6512 | C17orf27 |  |
| Ssc.16410.1.A1_at | 2.65 | 0.0007 | 0.6512 | OPRS1 | Opioid receptor, sigma 1 isoform 1; SR31747 binding protein 1; type I sigma receptor [Homo sapiens] |
| Ssc.3345.1.A1_at | 2.43 | 0.0007 | 0.6512 | MVK | Mevalonate kinase (MK) |
| Ssc.17343.1.S1_at | -2.01 | 0.0007 | 0.6512 | XLKD1 | Extracellular link domain containing 1; lymphatic vessel endothelial hyaluronan receptor 1; extracellular link domain-containing 1; hyaluronic acid receptor [Homo sapiens] |
| Ssc.16664.1.A1_at | -2.15 | 0.0007 | 0.6512 | BTEB1 | Transcription factor BTEB1 (Basic transcription element binding protein 1) (BTE-binding protein 1) (GC box binding protein 1) (Krueppel-like factor 9) |
| Ssc.12202.2.S1_at | 3.24 | 0.0009 | 0.6512 | FDPS | Farnesyl pyrophosphate synthetase (FPP synthetase) (FPS) (Farnesyl diphosphate synthetase) [Includes: Dimethylallyltranstransferase; Geranyltranstransferase |
| Ssc.29672.1.A1_at | -2.27 | 0.0010 | 0.6512 | NRXN1 | Neurexin 1-beta precursor (Neurexin I-beta) |
| Ssc.6418.1.S1_at | 4.25 | 0.0011 | 0.6512 | FDFT1 | Farnesyl-diphosphate farnesyltransferase (Squalene synthetase) (SQS) (SS) (FPP:FPP farnesyltransferase) |
| Ssc.19059.1.A1_at | -2.47 | 0.0011 | 0.6512 | AGTR1 | Type-1 angiotensin II receptor (AT1) (AT1AR) |
| Ssc.6714.1.A1_at | 3.41 | 0.0012 | 0.6512 | IDI1 | Isopentenyl-diphosphate delta-isomerase 1 (IPP isomerase 1) (Isopentenyl pyrophosphate isomerase 1) (IPPI1) |
| Ssc.11260.1.A1_at | 2.20 | 0.0013 | 0.6512 | GSTA1 | Glutathione S-transferase A1 (GTH1) (HA subunit 1) (GST- epsilon) (GSTA1-1) (GST class-alpha) |
| Ssc.5712.1.S1_at | 2.88 | 0.0014 | 0.6512 | CYP51A1 | Cytochrome P450 51A1 (CYPLI) (P450LI) (Sterol 14-alpha demethylase) (Lanosterol 14-alpha demethylase) (LDM) (P450-14DM) |
| Ssc.3703.1.S1_at | 15.43 | 0.0015 | 0.6512 | APOA2 | Apolipoprotein A-II precursor (Apo-AII) (ApoA-II) |
| Ssc.5455.1.S1_at | 3.84 | 0.0015 | 0.6512 | DHCR7 | 7-dehydrocholesterol reductase (7-DHC reductase) (Sterol delta-7-reductase) (Putative sterol reductase SR-2) |
| Ssc.13068.1.A1_at | 3.19 | 0.0017 | 0.6512 | ZC3HDC1 | Zinc finger CCCH type domain containing protein 1 |
| Ssc.4685.1.S1_at | 2.86 | 0.0017 | 0.6512 | MVD | Diphosphomevalonate decarboxylase (Mevalonate pyrophosphate decarboxylase) (Mevalonate (diphospho)decarboxylase) |
| Ssc.28450.1.S1_at | 2.30 | 0.0017 | 0.6512 | Q96F59 |  |
| Ssc.10606.1.S1_at | 4.22 | 0.0018 | 0.6512 | ZNF507 | Zinc finger protein 507 |
| Ssc.17337.1.S1_at | -3.56 | 0.0018 | 0.6512 | TLR2 | Toll-like receptor 2 precursor (Toll/interleukin 1 receptor-like protein 4) |
| Ssc.336.1.S1_at | 5.75 | 0.0020 | 0.6512 | USP18 | Ubl carboxyl-terminal hydrolase 18 (Ubl thiolesterase 18) (ISG15-specific processing protease) (43 kDa ISG15-specific protease) (hUBP43) |
| Ssc.16125.1.S1_x_at | 15.23 | 0.0022 | 0.6512 | MT1J | Metallothionein-IE (MT-1E) |
| Ssc.600.1.S1_s_at | 28.70 | 0.0023 | 0.6512 | MT1J | Metallothionein-IE (MT-1E) |
| Ssc.15727.1.A1_at | 2.34 | 0.0024 | 0.6512 | SC5DL | Lathosterol oxidase (Lathosterol 5-desaturase) (Delta-7- sterol 5-desaturase) (C-5 sterol desaturase) (Sterol-C5-desaturase) |
| Ssc.16114.1.S1_at | -3.48 | 0.0025 | 0.6512 | CACNA2D1 | Dihydropyridine-sensitive L-type, calcium channel alpha-2/delta subunits precursor |
| Ssc.8925.1.S1_at | -2.79 | 0.0028 | 0.6611 | HBA1 | Hemoglobin alpha chain |
| Ssc.21162.1.S1_s_at | 5.00 | 0.0029 | 0.6611 | IRF7 | Interferon regulatory factor 7 (IRF-7) |
| Ssc.12504.1.A1_at | 3.46 | 0.0029 | 0.6611 | FAM14A | Protein FAM14A precursor (TLH29 protein) (pIFI27-like protein) |
| Ssc.6582.3.A1_a_at | 2.10 | 0.0029 | 0.6611 | Q96CU9 |  |
| Ssc.26189.1.S1_a_at | 4.70 | 0.0031 | 0.6611 |  | 28 kDa protein |
| Ssc.7157.1.A1_at | -2.50 | 0.0031 | 0.6611 | HBB | Hemoglobin beta chain |
| Ssc.2641.1.S1_at | 3.86 | 0.0032 | 0.6611 | UBE2L6 | Ubiquitin-conjugating enzyme E2-18 kDa UbcH8 (Ubiquitin- protein ligase) (Ubiquitin carrier protein) (Retinoic acid induced gene B protein) (RIG-B) |
| Ssc.4426.2.S1_at | 6.37 | 0.0033 | 0.6611 | TCP1 | T-complex protein 1, alpha subunit (TCP-1-alpha) (CCT-alpha) |
| Ssc.16410.3.S1_at | 2.33 | 0.0033 | 0.6611 | OPRS1 | Opioid receptor, sigma 1 isoform 1; SR31747 binding protein 1; type I sigma receptor [Homo sapiens] |
| Ssc.20837.1.S1_at | -2.02 | 0.0038 | 0.6801 | Q9NQF0 |  |
| Ssc.18175.1.A1_at | 3.92 | 0.0041 | 0.6801 | FASN | Fatty acid synthase |
| Ssc.30373.1.A1_at | 2.11 | 0.0042 | 0.6801 | PDE5A | cGMP-specific 3',5'-cyclic phosphodiesterase (CGB-PDE) (cGMP-binding cGMP-specific phosphodiesterase) |
| Ssc.3271.1.A1_at | -2.61 | 0.0046 | 0.6920 | ABCA1 | ATP-binding cassette, sub-family A, member 1 (ATP-binding cassette transporter 1) (ATP-binding cassette 1) (ABC-1) (Cholesterol efflux regulatory protein) |
| Ssc.19298.2.S1_at | 2.75 | 0.0049 | 0.7052 | DHCR24 | 24-dehydrocholesterol reductase precursor (3-beta- hydroxysterol delta-24-reductase) (Seladin-1) (Diminuto/dwarf1 homolog) |
| Ssc.1121.1.S1_at | -3.19 | 0.0054 | 0.7135 | PDK4 | Pyruvate dehydrogenase kinase isoform 4 |
| Ssc.8774.1.S1_at | 3.35 | 0.0055 | 0.7135 | SC4MOL | C-4 methyl sterol oxidase |
| Ssc.26115.1.S1_at | 2.25 | 0.0055 | 0.7135 | Q5VVD4 | PREDICTED: similar to Hypothetical protein MGC37938 |
| Ssc.15695.1.S1_at | -7.28 | 0.0055 | 0.7135 | RBP4 | Plasma retinol-binding protein precursor (PRBP) (RBP) (PRO2222) |
| Ssc.4426.1.S1_at | 4.37 | 0.0059 | 0.7135 | TCP1 | T-complex protein 1, alpha subunit (TCP-1-alpha) (CCT-alpha) |
| Ssc.17889.1.A1_at | 3.91 | 0.0061 | 0.7135 | GBP2 | Interferon-induced guanylate-binding protein 2 (GTP-binding protein 2) (Guanine nucleotide-binding protein 2) (HuGBP-2) |
| Ssc.11163.1.S1_at | 2.81 | 0.0064 | 0.7203 | SP140 | LYSp100 protein (Lymphoid-restricted homolog of Sp100) (Nuclear autoantigen Sp-140) (Speckled 140 kDa) (Nuclear body protein Sp140) |
| Ssc.9939.1.A1_at | -3.02 | 0.0064 | 0.7203 | Q5T9X8 | Collagen, type IV, alpha 2 (Fragment) |
| Ssc.286.1.S1_s_at | 10.98 | 0.0065 | 0.7203 | NP_542388 | Viperin; similar to inflammatory response protein 6 [Homo sapiens] |
| Ssc.11126.1.A1_at | 2.96 | 0.0066 | 0.7203 | HMGCS1 | Hydroxymethylglutaryl-CoA synthase, cytoplasmic (HMG-CoA synthase) (3-hydroxy-3-methylglutaryl coenzyme A synthase) |
| Ssc.2173.1.A1_at | 2.74 | 0.0068 | 0.7218 | ADR2_HUMAN | Adiponectin receptor protein 2 (Progestin and adipoQ receptor family member II) |
| Ssc.27562.1.S1_at | -2.03 | 0.0068 | 0.7218 | DDX5 | Probable RNA-dependent helicase p68 (DEAD-box protein p68) (DEAD-box protein 5) |
| Ssc.3581.1.S1_at | 2.75 | 0.0070 | 0.7330 | DNPEP | Aspartyl aminopeptidase (EC 3.4.11.21) |
| Ssc.646.1.S1_at | -5.53 | 0.0072 | 0.7330 | CSTA | Cystatin A (Stefin A) (Cystatin AS) |
| Ssc.16377.1.A1_s_at | 3.31 | 0.0078 | 0.7330 | GSTA1 | Glutathione S-transferase A1 (GTH1) (HA subunit 1) (GST- epsilon) (GSTA1-1) (GST class-alpha) |
| AFFX-Ss_IRP_3_at | 8.28 | 0.0084 | 0.7330 |  |  |
| Ssc.10605.1.A1_at | -2.89 | 0.0084 | 0.7330 | MAP6 | Microtubule-associated protein 6 isoform 1; stable tubule-only polypeptide [Homo sapiens] |
| Ssc.10588.1.A1_at | 2.36 | 0.0086 | 0.7330 | C1orf29 | Histocompatibility 28 [Homo sapiens] |
| Ssc.15992.1.S1_at | 2.35 | 0.0088 | 0.7330 | SREBF1 | Sterol regulatory element binding protein-1 (SREBP-1) (Sterol regulatory element-binding transcription factor 1) |
| Ssc.18947.1.A1_at | 2.66 | 0.0089 | 0.7330 | SPINK5 | Serine protease inhibitor Kazal-type 5 precursor (Lympho-epithelial Kazal-type related inhibitor) (LEKTI) [Contains: Hemofiltrate peptide HF6478; Hemofiltrate peptide HF7665] |
| Ssc.4729.1.S2_at | 4.48 | 0.0091 | 0.7330 | Q7Z2T0 | MSTP111 |
| Ssc.3345.2.S1_at | 2.47 | 0.0091 | 0.7330 | MVK | Mevalonate kinase (MK) |
| Ssc.6528.1.S1_at | 2.60 | 0.0093 | 0.7330 | AGPAT2 | 1-acyl-sn-glycerol-3-phosphate acyltransferase beta (EC 2.3.1.51) (1- AGP acyltransferase 2) (1-AGPAT 2) (Lysophosphatidic acid acyltransferase-beta) (LPAAT-beta) (1-acylglycerol-3-phosphate O- acyltransferase 2) |
| Ssc.6850.1.A1_at | 2.25 | 0.0093 | 0.7330 | NANS | Sialic acid synthase (N-acetylneuraminate synthase) (N- acetylneuraminic acid synthase) (N-acetylneuraminate-9-phosphate synthase) (N-acetylneuraminic acid phosphate synthase) |
| Ssc.22588.2.S1_at | -2.84 | 0.0094 | 0.7330 | GABARAPL1 | Gamma-aminobutyric acid receptor-associated protein-like 1 (GABA(A) receptor-associated protein-like 1) (Glandular epithelial cell protein 1) (GEC-1) (Early estrogen-regulated protein) |
| Ssc.30724.1.S1_at | 3.19 | 0.0097 | 0.7330 | HERC6 | Hect domain and RLD 6 [Homo sapiens] |

**Additional file 2B**

| **Day 28 IR vs OUT** | | | | | |
| --- | --- | --- | --- | --- | --- |
| **affy.id** | **FC** | **P-value** | **Q-value** | **Gene Name** | **Product** |
| Ssc.3753.1.S1_at | 3.50 | 0.0000 | 0.0070 | TFRC | Transferrin receptor protein 1 (TfR1) (TR) (TfR) (Trfr) (CD71 antigen) (T9) (p90) |
| Ssc.5455.1.S1_at | 6.54 | 0.0000 | 0.0926 | DHCR7 | 7-dehydrocholesterol reductase (7-DHC reductase) (Sterol delta-7-reductase) (Putative sterol reductase SR-2) |
| Ssc.11875.1.A1_at | -2.26 | 0.0000 | 0.0926 | Q5VXM1 |  |
| Ssc.1121.1.S1_at | -11.62 | 0.0000 | 0.0995 | PDK4 | Pyruvate dehydrogenase kinase isoform 4 |
| Ssc.18175.1.A1_at | 3.47 | 0.0001 | 0.1666 | FASN | Fatty acid synthase |
| Ssc.5455.2.S1_at | 3.36 | 0.0001 | 0.1666 | DHCR7 | 7-dehydrocholesterol reductase (7-DHC reductase) (Sterol delta-7-reductase) (Putative sterol reductase SR-2) |
| Ssc.17718.1.S1_at | 2.57 | 0.0001 | 0.1666 | IGHA2 | Ig alpha-2 chain C region. |
| Ssc.6433.1.S1_at | 2.37 | 0.0001 | 0.1666 | Q9P2E3 |  |
| Ssc.30870.1.A1_at | 2.36 | 0.0001 | 0.1666 | NP_859077 | Low density lipoprotein receptor-related protein binding protein [Homo sapiens] |
| Ssc.12596.1.A1_at | 2.15 | 0.0001 | 0.1666 | RORC | Nuclear receptor ROR-gamma (Nuclear receptor RZR-gamma) |
| Ssc.30512.1.A1_at | -2.34 | 0.0001 | 0.1666 | CA7 | Carbonic anhydrase VII (Carbonate dehydratase VII) (CA-VII) |
| Ssc.229.1.S1_at | -3.25 | 0.0001 | 0.1666 | FMO1 | Dimethylaniline monooxygenase [N-oxide-forming] 1(Fetal hepatic flavin-containing monooxygenase 1) (FMO 1) (Dimethylaniline oxidase 1) |
| Ssc.10131.1.A1_at | -8.44 | 0.0001 | 0.1666 | PDK4 | Pyruvate dehydrogenase kinase isoform 4 |
| Ssc.27155.1.S1_at | -3.34 | 0.0001 | 0.1918 | SLC39A4 | Solute carrier family 39 (zinc transporter), member 4; acrodermatitis enteropathica, zinc-deficiency type [Homo sapiens] |
| Ssc.27786.1.S1_at | 3.55 | 0.0002 | 0.1918 | PFKFB3 | 6-phosphofructo-2-kinase/fructose-2,6-biphosphatase 3 (6PF-2-K/Fru- 2,6-P2ASE brain/placenta-type isozyme) (iPFK-2) [Includes: 6- phosphofructo-2-kinase (EC 2.7.1.105); Fructose-2,6-bisphosphatase |
| Ssc.331.2.S1_at | 2.00 | 0.0002 | 0.1918 | LDLR | Low-density lipoprotein receptor precursor (LDL receptor) |
| Ssc.38.1.S1_at | 6.88 | 0.0002 | 0.1989 | TFRC | Transferrin receptor protein 1 (TfR1) (TR) (TfR) (Trfr) (CD71 antigen) (T9) (p90) |
| Ssc.657.1.A1_at | 3.49 | 0.0002 | 0.1989 | CCL2 | Small inducible cytokine A2 precursor (CCL2) (Monocyte chemotactic protein 1) (MCP-1) (Monocyte chemoattractant protein-1) (Monocyte chemotactic and activating factor) (MCAF) (Monocyte secretory protein JE) (HC11) |
| Ssc.17769.1.S1_at | 2.10 | 0.0002 | 0.1989 | ACACA | Acetyl-CoA carboxylase 1 (EC 6.4.1.2) (ACC-alpha) [Includes: Biotin carboxylase] |
| Ssc.16159.1.S1_at | 11.88 | 0.0003 | 0.1989 | SCD | Acyl-CoA desaturase (Stearoyl-CoA desaturase) (Fatty acid desaturase) (Delta(9)-desaturase) |
| Ssc.26063.1.S1_at | 10.37 | 0.0003 | 0.1989 | INSIG1 | Insulin-induced protein 1 |
| Ssc.16125.1.S1_x_at | 5.57 | 0.0003 | 0.1989 | MT1J | Metallothionein-IE (MT-1E) |
| Ssc.15598.1.S1_at | 4.04 | 0.0003 | 0.1989 | INSIG1 | Insulin-induced protein 1 |
| Ssc.12918.1.S1_at | 2.39 | 0.0003 | 0.1989 | NMI | N-myc-interactor (Nmi) (N-myc and STAT interactor) |
| SscAffx.1.1.S1_at | 2.33 | 0.0003 | 0.1989 | ISG20 | Interferon stimulated gene 20kDa; interferon stimulated gene (20kD) [Homo sapiens] |
| Ssc.9957.1.A1_at | 4.41 | 0.0004 | 0.2155 | CCL8 | Small inducible cytokine A8 precursor (CCL8) (Monocyte chemotactic protein 2) (MCP-2) (Monocyte chemoattractant protein 2) (HC14) [Contains: MCP-2(6-76)] |
| Ssc.16160.1.S1_at | 3.80 | 0.0004 | 0.2155 | CD86 | T lymphocyte activation antigen CD86 precursor (Activation B7-2 antigen) (CTLA-4 counter-receptor B7.2) (B70) (FUN-1) (BU63) |
| Ssc.18990.1.A1_at | 3.10 | 0.0004 | 0.2155 | FRMD3 | FERM domain containing 3; band 4.1-like protein 4; protein 4.1O [Homo sapiens] |
| Ssc.8790.1.A1_at | 4.16 | 0.0004 | 0.2227 | CCL28 | Small inducible cytokine A28 precursor (CCL28) (Mucosae-associated epithelial chemokine) (MEC) (CCK1 protein) |
| Ssc.8385.1.A1_at | 6.34 | 0.0005 | 0.2377 | SQLE | Squalene monooxygenase (Squalene epoxidase) (SE) |
| Ssc.8774.1.S1_at | 3.53 | 0.0005 | 0.2377 | SC4MOL | C-4 methyl sterol oxidase |
| Ssc.1674.1.A1_at | 2.82 | 0.0005 | 0.2377 | SLC2A14 | Glucose transporter 14 [Homo sapiens] |
| Ssc.28336.1.A1_at | -2.23 | 0.0005 | 0.2377 | SH3BP5 | SH3 domain-binding protein 5 (SH3 domain-binding protein that preferentially associates with BTK) |
| Ssc.9198.1.S1_at | -3.26 | 0.0005 | 0.2377 | SAH3_HUMAN | Putative adenosylhomocysteinase 3 (S-adenosyl-L- homocysteine hydrolase) (AdoHcyase) |
| Ssc.5712.1.S1_at | 2.64 | 0.0006 | 0.2377 | CYP51A1 | Cytochrome P450 51A1 (CYPLI) (P450LI) (Sterol 14-alpha demethylase) (Lanosterol 14-alpha demethylase) (LDM) (P450-14DM) |
| Ssc.25282.1.S1_at | -2.00 | 0.0006 | 0.2377 | SH3BP5 | SH3 domain-binding protein 5 (SH3 domain-binding protein that preferentially associates with BTK) |
| Ssc.4685.1.S1_at | 2.51 | 0.0006 | 0.2381 | MVD | Diphosphomevalonate decarboxylase (Mevalonate pyrophosphate decarboxylase) (Mevalonate (diphospho)decarboxylase) |
| Ssc.18553.1.S1_at | 2.07 | 0.0006 | 0.2381 | HLA-B | HLA class I histocompatibility antigen, B-7 alpha chain precursor (MHC class I antigen B*7) |
| Ssc.11126.1.A1_at | 2.89 | 0.0006 | 0.2550 | HMGCS1 | Hydroxymethylglutaryl-CoA synthase, cytoplasmic (HMG-CoA synthase) (3-hydroxy-3-methylglutaryl coenzyme A synthase) |
| Ssc.6168.1.S1_at | 2.65 | 0.0007 | 0.2588 | ETV7 | Transcription factor ETV7 (Transcription factor Tel-2) (ETS-related protein Tel2) (Tel related Ets factor) |
| Ssc.4426.1.S1_at | 3.99 | 0.0008 | 0.2871 | TCP1 | T-complex protein 1, alpha subunit (TCP-1-alpha) (CCT-alpha) |
| Ssc.12456.1.A1_at | 3.13 | 0.0008 | 0.2871 | NP_115821 | MEGF11 protein [Homo sapiens] |
| Ssc.1217.1.S1_at | 2.01 | 0.0008 | 0.2871 | PHF11 | PHD finger protein 11 (BRCA1-C terminus associated protein) (NY-REN-34 antigen) |
| Ssc.315.1.S1_at | -7.35 | 0.0008 | 0.2871 | DPEP1 | Microsomal dipeptidase precursor (MDP) (Dehydropeptidase-I) (Renal dipeptidase) (RDP) |
| Ssc.15727.1.A1_at | 2.97 | 0.0009 | 0.2871 | SC5DL | Lathosterol oxidase (Lathosterol 5-desaturase) (Delta-7- sterol 5-desaturase) (C-5 sterol desaturase) (Sterol-C5-desaturase) |
| Ssc.16088.1.S1_at | 2.36 | 0.0010 | 0.2985 | HMGCR | 3-hydroxy-3-methylglutaryl-coenzyme A reductase (HMG-CoA reductase) |
| Ssc.4426.2.S1_at | 4.50 | 0.0011 | 0.3086 | TCP1 | T-complex protein 1, alpha subunit (TCP-1-alpha) (CCT-alpha) |
| Ssc.11108.1.A1_at | -2.40 | 0.0011 | 0.3086 | PMP22 | Peripheral myelin protein 22 (PMP-22) |
| Ssc.11006.1.S1_at | 2.68 | 0.0012 | 0.3159 | SNX10 | Sorting nexin 10 |
| Ssc.6714.1.A1_at | 2.60 | 0.0012 | 0.3159 | IDI1 | Isopentenyl-diphosphate delta-isomerase 1 (IPP isomerase 1) (Isopentenyl pyrophosphate isomerase 1) (IPPI1) |
| Ssc.15640.1.S1_at | 7.55 | 0.0012 | 0.3216 | MT2A | Metallothionein-II (MT-II) (Metallothionein 2A) |
| Ssc.600.1.S1_s_at | 13.01 | 0.0014 | 0.3218 | MT1J | Metallothionein-IE (MT-1E) |
| Ssc.12795.1.S1_at | 2.21 | 0.0014 | 0.3218 | TREX1 | ATR-interacting protein (ATM and Rad3 related interacting protein) |
| Ssc.12202.2.S1_at | 2.76 | 0.0015 | 0.3218 | FDPS | Farnesyl pyrophosphate synthetase (FPP synthetase) (FPS) (Farnesyl diphosphate synthetase) [Includes: Dimethylallyltranstransferase; Geranyltranstransferase |
| Ssc.21302.2.S1_at | 2.19 | 0.0015 | 0.3218 | ENPP6 | ectonucleotide pyrophosphatase/phosphodiesterase 6; B830047L21Rik [Homo sapiens] |
| Ssc.30963.1.A1_at | -2.26 | 0.0017 | 0.3224 | SLC6A4 | Sodium-dependent serotonin transporter (5HT transporter) (5HTT) |
| Ssc.4152.1.A1_at | -3.92 | 0.0017 | 0.3224 | LRRC15 | 18 kDa protein |
| Ssc.37.1.S1_at | 2.79 | 0.0019 | 0.3497 | HPR | Haptoglobin-related protein precursor |
| Ssc.26005.1.S1_at | 3.29 | 0.0020 | 0.3545 | ZBP1 | Z-DNA binding protein 1 (Tumor stroma and activated macrophage protei DLM-1) |
| Ssc.31140.1.S1_at | 2.18 | 0.0022 | 0.3733 | IFIT3 | Interferon-induced protein with tetratricopeptide repeats 3 (IFIT-3) (IFIT-4) (Interferon-induced 60 kDa protein) (IFI-60K) (ISG-60) (CIG49) (Retinoic acid-induced gene G protein) (RIG-G) |
| Ssc.23510.1.A1_s_at | 2.20 | 0.0024 | 0.3759 | HLA-G | HLA class I histocompatibility antigen, alpha chain G precursor (HLA G antigen) |
| Ssc.11098.1.S1_at | 2.63 | 0.0025 | 0.3839 | IFITM3 | Interferon-induced transmembrane protein 3 (Interferon-inducible protein 1-8U) |
| Ssc.26328.1.S1_at | 2.06 | 0.0025 | 0.3839 | CCR5 | C-C chemokine receptor type 5 (C-C CKR-5) (CC-CKR-5) (CCR-5) (CCR5) (HIV-1 fusion coreceptor) (CHEMR13) (CD195 antigen) |
| Ssc.23041.1.S1_at | 5.54 | 0.0025 | 0.3868 |  | 13 kDa protein |
| Ssc.19059.1.A1_at | -2.27 | 0.0026 | 0.3868 | AGTR1 | Type-1 angiotensin II receptor (AT1) (AT1AR) |
| Ssc.1093.2.A1_at | -2.41 | 0.0026 | 0.3868 | FZD4 | Frizzled 4 precursor (Frizzled-4) (Fz-4) (hFz4) (FzE4) |
| Ssc.11162.1.S1_at | 3.17 | 0.0028 | 0.3944 | C4BPA | C4b-binding protein alpha chain precursor (C4bp) (Proline-rich protein) (PRP) |
| Ssc.15674.1.A1_at | 2.38 | 0.0029 | 0.4046 | CTSL | Cathepsin L precursor (Major excreted protein) (MEP) |
| Ssc.18096.1.A1_at | -3.00 | 0.0030 | 0.4046 | KCTD8 | Potassium channel tetramerisation domain containing 8 [Homo sapiens] |
| Ssc.15942.3.S1_x_at | 7.84 | 0.0034 | 0.4186 | IGHM | Ig alpha-1 chain C region |
| Ssc.9707.1.A1_at | 2.14 | 0.0034 | 0.4186 | BTG2 | BTG2 protein (NGF-inducible anti-proliferative protein PC3) |
| Ssc.11048.1.S1_at | 4.14 | 0.0035 | 0.4206 | PLAC8 | Placenta-specific gene 8 protein (C15 protein) (BM-004) |
| Ssc.6418.1.S1_at | 2.47 | 0.0036 | 0.4226 | FDFT1 | Farnesyl-diphosphate farnesyltransferase (Squalene synthetase) (SQS) (SS) (FPP:FPP farnesyltransferase) |
| Ssc.22000.1.S1_at | -2.75 | 0.0036 | 0.4226 | CBR1 | Carbonyl reductase [NADPH] 1 (NADPH-dependent carbonyl reductase 1) (Prostaglandin-E(2) 9-reductase) (Prostaglandin 9-ketoreductase) (15-hydroxyprostaglandin dehydrogenase [NADP+]) |
| Ssc.2635.1.S1_at | -2.80 | 0.0037 | 0.4251 | HSD11B2 | Corticosteroid 11-beta-dehydrogenase, isozyme 2 (11-DH2) (11-beta-hydroxysteroid dehydrogenase type 2) (11-beta-HSD2) (NAD- dependent 11-beta-hydroxysteroid dehydrogenase) |
| Ssc.8774.2.A1_at | 2.72 | 0.0038 | 0.4251 | SC4MOL | C-4 methyl sterol oxidase |
| Ssc.28870.1.S1_at | 2.19 | 0.0038 | 0.4251 |  |  |
| Ssc.10441.1.S1_at | -9.75 | 0.0039 | 0.4256 | UGT2B17 | UDP-glucuronosyltransferase 2B17 precursor, microsomal (UDPGT) (C19-steroid specific UDP-glucuronosyltransferase) |
| Ssc.11145.1.A1_at | 4.71 | 0.0040 | 0.4256 | CD5L | CD5 antigen-like precursor (SP-alpha) (CT-2) (IgM-associated peptide) |
| Ssc.5045.1.S1_at | 4.25 | 0.0045 | 0.4542 | EBP | 3-beta-hydroxysteroid-delta(8),delta(7)-isomerase (Cholestenol delta-isomerase) (Delta8-delta7 sterol isomerase) (D8-D7 sterol isomerase) (Emopamil-binding protein) |
| Ssc.25136.2.A1_a_at | -2.21 | 0.0049 | 0.4606 | CNDP1 | Glutamate carboxypeptidase-like protein 2 precursor (CNDP dipeptidase 1) |
| Ssc.21926.1.S1_at | 3.56 | 0.0053 | 0.4725 | LDLR | Low-density lipoprotein receptor precursor (LDL receptor) |
| Ssc.25317.1.S1_at | -7.43 | 0.0053 | 0.4725 | PDZK10 | PDZ domain containing 10 |
| Ssc.8980.1.A1_at | -2.35 | 0.0058 | 0.4834 | ANGPTL4 | Angiopoietin-related protein 4 precursor (Angiopoietin-like 4) (Hepatic fibrinogen/angiopoietin-related protein) (HFARP) (Angiopoietin-like protein PP1158) |
| Ssc.16937.1.A1_at | 2.06 | 0.0059 | 0.4834 | TP53INP2 | Tumor protein p53 inducible nuclear protein 2 |
| Ssc.5022.1.A1_at | -3.21 | 0.0062 | 0.4880 | NP_114162 | NYD-SP14 protein [Homo sapiens] |
| Ssc.25739.1.S1_at | 3.56 | 0.0062 | 0.4883 | IRF7 | Interferon regulatory factor 7 (IRF-7) |
| Ssc.11073.1.S1_at | 2.07 | 0.0065 | 0.4894 | PSMB8 | Proteasome subunit beta type 8 precursor (Proteasome component C13) (Macropain subunit C13) (Multicatalytic endopeptidase complex subunit C13) |
| Ssc.22620.1.S1_at | 3.16 | 0.0066 | 0.4894 | IFIT2 | Interferon-induced protein with tetratricopeptide repeats 2 (IFIT-2) (Interferon-induced 54 kDa protein) (IFI-54K) (ISG-54 K) |
| Ssc.12286.2.A1_at | 2.22 | 0.0067 | 0.4894 | PML | Probable transcription factor PML (Tripartite motif protein 19) |
| Ssc.6433.2.S1_at | 2.70 | 0.0068 | 0.4894 | Q9P2E3 |  |
| Ssc.220.1.A1_at | 4.44 | 0.0071 | 0.4894 | MX2 | Interferon-regulated resistance GTP-binding protein MxB (p78-related protein) |
| Ssc.15942.2.S1_x_at | 8.36 | 0.0073 | 0.4917 | IGHM | Ig alpha-1 chain C region |
| Ssc.26146.1.S1_at | 6.28 | 0.0074 | 0.4948 | CXCL9 | Small inducible cytokine B9 precursor (CXCL9) (Gamma interferon induced monokine) (MIG) |
| Ssc.13778.1.S1_at | 5.17 | 0.0075 | 0.4963 | IGHM | Ig alpha-1 chain C region |
| Ssc.19659.2.S1_at | 2.09 | 0.0080 | 0.5036 | NP_005759 | Gene rich cluster, C3f gene [Homo sapiens] |
| Ssc.9229.1.S1_at | 2.19 | 0.0081 | 0.5036 | MRC1 | Macrophage mannose receptor precursor (MMR) (CD206 antigen) |
| Ssc.29092.1.A1_at | 2.12 | 0.0081 | 0.5036 | KRAS2 | Transforming protein p21 (K-Ras 2) (Ki-Ras) (c-K-ras) |
| Ssc.2290.1.S1_at | -2.02 | 0.0082 | 0.5036 | O94958 |  |
| Ssc.7290.1.A1_at | -2.39 | 0.0082 | 0.5036 | C1QDC1 | C1q domain containing 1 isoform 2 [Homo sapiens] |
| Ssc.1093.3.S1_at | -2.64 | 0.0082 | 0.5036 | FZD4 | Frizzled 4 precursor (Frizzled-4) (Fz-4) (hFz4) (FzE4) |
| Ssc.13128.1.A1_at | 2.08 | 0.0083 | 0.5036 | Q8IY21 |  |
| Ssc.646.1.S1_at | -6.90 | 0.0083 | 0.5036 | CSTA | Cystatin A (Stefin A) (Cystatin AS) |
| Ssc.26009.1.S1_at | 2.55 | 0.0086 | 0.5160 | HLCS | Biotin--protein ligase (Biotin apo-protein ligase) [Includes: Biotin--[methylmalonyl-CoA-carboxytransferase] ligase; Biotin--[propionyl-CoA-carboxylase [ATP-hydrolyzing]] ligase (Holocarboxylase synthetase) (HCS); Biotin-- [methylcrotonoyl-CoA-carboxylase] ligase Biotin-- [acetyl-CoA-carboxylase] ligase] |
| Ssc.4914.1.A1_at | -2.90 | 0.0086 | 0.5160 | Q9H8M9 |  |
| Ssc.12286.1.A1_at | 2.19 | 0.0087 | 0.5160 | PML | Probable transcription factor PML (Tripartite motif protein 19) |
| Ssc.11557.1.A1_at | 2.48 | 0.0091 | 0.5244 | G1P2 | Ubiquitin cross-reactive protein precursor (Interferon-induced 17 kDa protein) (Interferon-induced 15 kDa protein) |
| Ssc.6676.1.S1_at | 3.30 | 0.0091 | 0.5264 | SL2B_HUMAN | Slp homolog lacking C2 domains-b (Exophilin 5) |
| Ssc.17894.1.A1_at | 2.15 | 0.0097 | 0.5281 | IFIH1 | Melanoma differentiation associated protein-5; DEAD/H (Asp-Glu-Ala-Asp/His) box polypeptide [Homo sapiens] |
| Ssc.23797.1.S1_at | 2.33 | 0.0099 | 0.5281 | CCL4L | Chemokine (C-C motif) ligand 4-like precursor; small inducible cytokine 4-like; small inducible cytokine A4-like; lymphocyte activation gene 1; macrophage inflammatory protein-1b2 [Homo sapiens] |
| Ssc.17889.1.A1_at | 2.24 | 0.0100 | 0.5281 | GBP2 | Interferon-induced guanylate-binding protein 2 (GTP-binding protein 2) (Guanine nucleotide-binding protein 2) (HuGBP-2) |

**Additional file 2C**

| **Day 56 IR vs OUT** | | | | | |
| --- | --- | --- | --- | --- | --- |
| **affy.id** | **FC** | **P-value** | **Q-value** | **Gene Name** | **Product** |
| Ssc.16718.1.A1_at | 3.18 | 0.0001 | 0.5265 | CTBS | Di-N-acetylchitobiase precursor |
| Ssc.27596.1.S1_at | 4.14 | 0.0004 | 0.5265 | AQP1 | Aquaporin-CHIP (Water channel protein for red blood cells and kidney proximal tubule) (Aquaporin 1) (AQP-1) (Urine water channel) |
| Ssc.24194.1.S1_a_at | 2.61 | 0.0004 | 0.5265 | TCEA2 | Transcription elongation factor A protein 2 (Transcription elongation factor S-II protein 2) (Testis-specific S-II) (Transcription elongation factor TFIIS.l) |
| Ssc.19629.1.A1_at | -2.79 | 0.0005 | 0.5265 | EGR1 | Early growth response protein 1 (EGR-1) (Krox-24 protein) (ZIF268) (Nerve growth factor-induced protein A) (NGFI-A) (Transcription factor ETR103) (Zinc finger protein 225) (AT225) |
| Ssc.14490.1.S1_at | 2.02 | 0.0006 | 0.5265 | LTB4DH | NADP-dependent leukotriene B4 12-hydroxydehydrogenase |
| Ssc.2364.1.S1_at | 2.88 | 0.0007 | 0.5265 | PER3 | Period circadian protein 3 (hPER3) |
| Ssc.23222.1.S1_at | 2.29 | 0.0007 | 0.5265 | PDXP | Pyridoxal phosphate phosphatase |
| Ssc.12642.1.S1_at | 2.21 | 0.0007 | 0.5265 | TEF | Thyrotroph embryonic factor |
| Ssc.22075.3.A1_at | -3.20 | 0.0008 | 0.5265 | SELL | L-selectin precursor (Lymph node homing receptor) (Leukocyte adhesion molecule-1) (LAM-1) (Leukocyte surface antigen Leu-8) (TQ1) (gp90-MEL) (Leukocyte-endothelial cell adhesion molecule 1) (LECAM1) (CD62L) |
| Ssc.15250.2.S1_at | 2.20 | 0.0010 | 0.5265 | KAI1 | CD82 antigen (Inducible membrane protein R2) (C33 antigen) (IA4) (Metastasis suppressor Kangai 1) (Suppressor of tumorigenicity-6) |
| Ssc.4466.1.S1_at | -2.10 | 0.0011 | 0.5265 | TEC | Tyrosine-protein kinase Tec |
| Ssc.10131.1.A1_at | -3.67 | 0.0011 | 0.5265 | PDK4 | [Pyruvate dehydrogenase [lipoamide]] kinase isozyme 4, mitochondrial precursor (EC 2.7.1.99) (Pyruvate dehydrogenase kinase isoform 4) |
| Ssc.19132.1.A1_at | 3.74 | 0.0013 | 0.5265 | EMP1 | Epithelial membrane protein-1 (EMP-1) (Tumor-associated membrane protein) (CL-20) (B4B protein) |
| Ssc.21905.1.S1_at | 2.97 | 0.0013 | 0.5265 | G6PU_HUMAN | Glucose 6-phosphate translocase (Glucose 5-phosphate transporter) (Solute carrier family 37 member 4) (PRO0685) |
| Ssc.19174.1.A1_at | 3.05 | 0.0014 | 0.5265 | PER2 | Period circadian protein 2 |
| Ssc.15565.1.S1_at | -2.36 | 0.0014 | 0.5265 | LCP2 | Lymphocyte cytosolic protein 2 (SH2 domain-containing leucocyte protein of 76 kDa) (SLP-76 tyrosine phosphoprotein) (SLP76) |
| Ssc.5704.1.S1_at | 3.00 | 0.0015 | 0.5265 | TNA | Tetranectin precursor (TN) (Plasminogen-kringle 4 binding protein) |
| Ssc.22470.1.S1_at | 2.22 | 0.0015 | 0.5265 | PER3 | Period circadian protein 3 (hPER3) |
| Ssc.9772.1.S1_at | -2.16 | 0.0017 | 0.5265 | RSBN1L | Round spermatid basic protein 1-like [Homo sapiens] |
| Ssc.22075.2.A1_at | -2.67 | 0.0017 | 0.5265 | SELL | L-selectin precursor (Lymph node homing receptor) (Leukocyte adhesion molecule-1) (LAM-1) (Leukocyte surface antigen Leu-8) (TQ1) (gp90-MEL) (Leukocyte-endothelial cell adhesion molecule 1) (LECAM1) (CD62L) |
| Ssc.15248.2.S1_at | 2.34 | 0.0018 | 0.5265 | APLP2 | Amyloid-like protein 2 precursor (Amyloid protein homolog) (APPH) (CDEI-box binding protein) (CDEBP) |
| Ssc.19571.2.S1_at | 2.04 | 0.0018 | 0.5265 | ABHD4 | Abhydrolase domain containing 4 [Homo sapiens] |
| Ssc.180.1.S1_at | -2.18 | 0.0018 | 0.5265 | TRGV9 | T-cell receptor gamma chain V region PT-gamma-1/2 precursor |
| Ssc.26321.1.S1_s_at | 2.05 | 0.0019 | 0.5265 | CYP2C18 | Cytochrome P450 2C18 (CYPIIC18) (P450-6B/29C) |
| Ssc.26431.1.A1_at | 2.53 | 0.0020 | 0.5265 | MYO1D | Myosin Id |
| Ssc.25372.1.S1_at | -2.33 | 0.0021 | 0.5265 | PIGR | Polymeric-immunoglobulin receptor precursor (Poly-Ig receptor) (PIGR) [Contains: Secretory component] |
| Ssc.15991.1.S1_at | 2.65 | 0.0024 | 0.5380 | CEBPB | CCAAT/enhancer binding protein beta (C/EBP beta) (Nuclear factor NF- IL6) (Transcription factor 5) |
| Ssc.206.1.S1_at | 2.25 | 0.0024 | 0.5380 | CYP2C18 | Cytochrome P450 2C18 (CYPIIC18) (P450-6B/29C) |
| Ssc.19365.1.S1_at | -2.02 | 0.0028 | 0.5380 | Q5R3K3 | PREDICTED: similar to gene; RIKEN cDNA 2810048G17 |
| Ssc.24975.1.S1_at | 2.55 | 0.0030 | 0.5380 | COL1A2 | Collagen alpha 2(I) chain precursor |
| Ssc.29972.1.A1_at | -2.80 | 0.0030 | 0.5380 | FAM31B |  |
| Ssc.4152.1.A1_at | 3.00 | 0.0031 | 0.5380 | LRRC15 | 18 kDa protein |
| Ssc.2697.1.S1_at | 2.62 | 0.0031 | 0.5380 | NP_060887 | Soluble adenylyl cyclase; adenylate cyclase; ATP pyrophosphate-lyase; 3',5'-cyclic AMP synthetase; AH-related protein; Hypercalciuria, absorptive, 2 [Homo sapiens] |
| Ssc.22731.1.S1_at | 2.07 | 0.0031 | 0.5380 | SEC14L2 | SEC14-like protein 2 (Alpha-tocopherol associated protein) (TAP) (hTAP) (Supernatant protein factor) (SPF) (Squalene transfer protein) |
| Ssc.4008.1.S1_at | -2.10 | 0.0031 | 0.5380 | C5orf18 | Polyposis locus protein 1 (TB2 protein) |
| Ssc.26266.1.S1_at | -2.53 | 0.0031 | 0.5380 | TYBN_HUMAN | NB thymosin beta |
| Ssc.12776.1.A1_at | -4.03 | 0.0031 | 0.5380 | SELL | L-selectin precursor (Lymph node homing receptor) (Leukocyte adhesion molecule-1) (LAM-1) (Leukocyte surface antigen Leu-8) (TQ1) (gp90-MEL) (Leukocyte-endothelial cell adhesion molecule 1) (LECAM1) (CD62L) |
| Ssc.2698.1.S1_at | 2.21 | 0.0032 | 0.5395 | FAAH | Fatty-acid amide hydrolase (Oleamide hydrolase) (Anandamide amidohydrolase) |
| Ssc.22033.3.S1_at | 2.11 | 0.0035 | 0.5451 | ACP2 | Lysosomal acid phosphatase precursor (LAP) |
| Ssc.13280.1.A1_at | -2.01 | 0.0036 | 0.5451 | RSBN1L | Round spermatid basic protein 1-like [Homo sapiens] |
| Ssc.22406.1.A1_at | 2.17 | 0.0039 | 0.5451 | AQP1 | Aquaporin-CHIP (Water channel protein for red blood cells and kidney proximal tubule) (Aquaporin 1) (AQP-1) (Urine water channel) |
| Ssc.16006.1.S1_at | -2.48 | 0.0047 | 0.5451 | PIGR | Polymeric-immunoglobulin receptor precursor (Poly-Ig receptor) (PIGR) [Contains: Secretory component] |
| Ssc.25550.1.S1_at | -3.02 | 0.0047 | 0.5451 | LY96 | Lymphocyte antigen 96 precursor (MD-2 protein) (ESOP-1) |
| Ssc.23538.1.S1_at | 2.12 | 0.0051 | 0.5451 | FMO5 | Dimethylaniline monooxygenase [N-oxide-forming] 5 (Hepatic flavin-containing monooxygenase 5) (FMO 5) (Dimethylaniline oxidase 5) |
| Ssc.24128.1.A1_at | -2.09 | 0.0053 | 0.5451 | O75458 | 20 kDa protein |
| Ssc.23489.1.S1_at | -2.40 | 0.0054 | 0.5451 | CD8A | T-cell surface glycoprotein CD8 alpha chain precursor (T-lymphocyte differentiation antigen T8/Leu-2) |
| Ssc.18802.1.A1_at | -2.66 | 0.0055 | 0.5451 | STX11 | Syntaxin 11 |
| Ssc.22075.1.S1_at | -2.86 | 0.0057 | 0.5451 | SELL | L-selectin precursor (Lymph node homing receptor) (Leukocyte adhesion molecule-1) (LAM-1) (Leukocyte surface antigen Leu-8) (TQ1) (gp90-MEL) (Leukocyte-endothelial cell adhesion molecule 1) (LECAM1) (CD62L) |
| Ssc.5901.2.S1_at | 2.03 | 0.0059 | 0.5530 | NP_060631 | NAD synthetase 1; glutamine-dependent NAD synthetase [Homo sapiens] |
| Ssc.16617.2.S1_at | 2.63 | 0.0060 | 0.5593 | PARK2 | Parkin isoform 1; parkin [Homo sapiens] |
| Ssc.26146.1.S1_at | -3.08 | 0.0064 | 0.5795 | CXCL9 | Small inducible cytokine B9 precursor (CXCL9) (Gamma interferon induced monokine) (MIG) |
| Ssc.1121.1.S1_at | -3.82 | 0.0067 | 0.5829 | PDK4 | Pyruvate dehydrogenase kinase isoform 4 |
| Ssc.11109.1.S1_at | -2.07 | 0.0075 | 0.5926 | PIK3CG | Phosphatidylinositol-4,5-bisphosphate 3-kinase catalytic subunit, gamma isoform (PI3-kinase p110 subunit gamma) (PtdIns- 3-kinase p110) (PI3K) (PI3Kgamma) |
| Ssc.22705.1.A1_at | -2.12 | 0.0075 | 0.5926 | MBNL1 | Muscleblind-like protein (Triplet-expansion RNA-binding protein) |
| Ssc.15850.1.S1_a_at | -3.47 | 0.0075 | 0.5926 | TCA_HUMAN | T-cell receptor alpha chain C region |
| Ssc.10620.1.A1_at | -2.42 | 0.0079 | 0.5926 | Q9P2H7 |  |
| Ssc.4804.1.S1_at | 2.90 | 0.0085 | 0.5926 | AGT | Angiotensinogen precursor [Contains: Angiotensin I (Ang I); Angiotensin II (Ang II); Angiotensin III (Ang III) (Des-Asp[1]- angiotensin II)] |
| Ssc.4632.2.A1_at | -2.00 | 0.0085 | 0.5926 | ARHGEF3 | Rho guanine nucleotide exchange factor 3; exchange factor found in platelets and leukemic and neuronal tissues, XPLN; RhoGEF protein; 59.8 kDA protein [Homo sapiens] |
| Ssc.9330.1.A1_at | -2.39 | 0.0086 | 0.5926 | LCP1 | L-plastin (Lymphocyte cytosolic protein 1) (LCP-1) (LC64P) |
| Ssc.15374.1.S1_at | 2.59 | 0.0087 | 0.5926 | COL14A1 | COL14A1 protein |
| Ssc.25227.1.S1_at | -2.07 | 0.0087 | 0.5926 | ARNTL | Aryl hydrocarbon receptor nuclear translocator-like protein 1 (Brain and muscle ARNT-like 1) (Member of PAS protein 3) (Basic-helix-loop- helix-PAS orphan MOP3) (bHLH-PAS protein JAP3) |
| Ssc.18707.1.A1_at | 2.12 | 0.0089 | 0.5926 | CTTNBP2 | Cortactin binding protein 2; cortactin-binding protein 2; chromosome 7 open reading frame 8 [Homo sapiens] |
| Ssc.428.10.S1_s_at | -3.07 | 0.0090 | 0.5926 | TCA_HUMAN | T-cell receptor alpha chain C region |
| Ssc.23519.1.S1_at | 2.02 | 0.0093 | 0.5926 | PLOD | Procollagen-lysine,2-oxoglutarate 5-dioxygenase 1 precursor (Lysyl hydroxylase 1) (LH1) |
| Ssc.11744.1.A1_at | -2.20 | 0.0096 | 0.5926 | NP_653321 | Multiple coiled-coil GABABR1-binding protein [Homo sapiens] |
| Ssc.18388.1.A1_at | -2.01 | 0.0097 | 0.5926 | COPG | Coatomer gamma subunit (Gamma-coat protein) (Gamma-COP) |

**Additional file 2D**

| **Day 5 IN vs OUT** | | | | | |
| --- | --- | --- | --- | --- | --- |
| **affy.id** | **FC** | **P-value** | **Q-value** | **Gene Name** | **Product** |
| Ssc.30869.1.S1_at | 2.78 | 0.0003 | 0.7553 | C17orf27 |  |
| Ssc.24849.1.S1_at | 2.19 | 0.0009 | 0.7553 | PCDH9 | Protocadherin 9 precursor |
| Ssc.26146.1.S1_at | 2.07 | 0.0038 | 0.7553 | CXCL9 | Small inducible cytokine B9 precursor (Gamma interferon induced monokine) (MIG) |
| Ssc.16114.1.S1_at | -2.23 | 0.0038 | 0.7553 | CACNA2D1 | Dihydropyridine-sensitive L-type, calcium channel alpha-2/delta subunits precursor |
| Ssc.1308.1.S1_at | 3.03 | 0.0039 | 0.7553 | GPC1 | Glypican-1 precursor. |
| Ssc.17934.1.S1_at | 3.68 | 0.0041 | 0.7553 | STARD10 | PCTP-like protein (StAR-related lipid transfer protein 10) (START domain-containing protein 10) (CGI-52) (Serologically defined colon cancer antigen 28) |
| Ssc.3753.1.S1_at | -5.01 | 0.0046 | 0.7553 | TFRC | Transferrin receptor protein 1 (CD71 antigen) (T9) (p90) |
| Ssc.16335.1.S2_at | -2.93 | 0.0053 | 0.7553 | LPL | Lipoprotein lipase precursor |
| Ssc.13504.1.A1_at | 3.02 | 0.0064 | 0.7553 | DHRS6 | Dehydrogenase/reductase (SDR family) member 6; oxidoreductase UCPA [Homo sapiens] |
| Ssc.396.1.S1_a_at | 2.30 | 0.0065 | 0.7553 | DBI | Acyl-CoA-binding protein (Diazepam binding inhibitor) (Endozepine) |
| Ssc.19327.1.S1_at | -2.24 | 0.0070 | 0.7553 | HIST1H1D | Histone H1.3 (Histone H1c) |
| Ssc.26189.1.S1_a_at | 2.18 | 0.0073 | 0.7553 |  | 28 kDa protein |
| Ssc.26005.1.S1_at | 2.00 | 0.0098 | 0.7553 | ZBP1 | Z-DNA binding protein 1 (Tumor stroma and activated macrophage protei DLM-1) |

**Additional file 2E**

| **Day 28 IN vs OUT** | | | | | |
| --- | --- | --- | --- | --- | --- |
| **affy.id** | **FC** | **P-value** | **Q-value** | **Gene Name** | **Product** |
| Ssc.2159.1.S1_at | -2.89 | 0.0002 | 0.8898 | OVOL1 | Putative transcription factor Ovo-like 1 (hOvo1) (Fragment) |
| Ssc.4690.1.S1_at | -2.03 | 0.0012 | 0.8898 | C9orf88 | Niban-like protein (Meg-3) |
| Ssc.26253.1.S1_at | -2.54 | 0.0017 | 0.8898 | LZTFL1 | Leucine zipper transcription factor-like 1 [Homo sapiens] |
| Ssc.5008.1.A1_at | 2.20 | 0.0019 | 0.8898 | GSTA4 | Glutathione S-transferase A4-4 (GST class-alpha) |
| Ssc.10078.1.A1_at | -2.70 | 0.0021 | 0.8898 | PCSK5 | Proprotein convertase subtilisin/kexin type 5 precursor (Proprotein convertase PC5) (Subtilisin/kexin-like protease PC5) (PC6) (hPC6) |
| Ssc.6019.1.S1_at | 2.12 | 0.0022 | 0.8898 | DNASE2 | Deoxyribonuclease II alpha precursor (DNase II alpha) (Acid DNase) (Lysosomal DNase II) (R31240_2) |
| Ssc.11108.1.A1_at | -2.87 | 0.0022 | 0.8898 | PMP22 | Peripheral myelin protein 22 (PMP-22) |
| Ssc.6165.1.A1_at | 3.93 | 0.0023 | 0.8898 | Q7L4P6 |  |
| Ssc.12157.1.A1_at | -2.56 | 0.0023 | 0.8898 | MYH14 | Myosin, heavy polypeptide 14; nonmuscle myosin heavy chain II-C; myosin heavy chain 14 [Homo sapiens] |
| Ssc.1310.1.S1_at | -2.06 | 0.0028 | 0.8898 | PTGES | Prostaglandin E synthase (Microsomal glutathione S- transferase 1-like 1) (MGST1-L1) (p53-induced apoptosis protein 12) |
| Ssc.11117.1.A1_at | -2.23 | 0.0030 | 0.8898 | PDE1B | Calcium/calmodulin-dependent 3',5'-cyclic nucleotide phosphodiesterase 1B (Cam-PDE 1B) (63 kDa Cam-PDE) |
| Ssc.18244.1.S1_at | 2.32 | 0.0032 | 0.8898 | NP_848634 | TAFA2 protein [Homo sapiens] |
| Ssc.25103.1.S1_at | -2.53 | 0.0034 | 0.8898 | ABCG2 | ATP-binding cassette, sub-family G, member 2 (Placenta-specific ATP- binding cassette transporter) (Breast cancer resistance protein) |
| Ssc.298.1.S1_at | -2.98 | 0.0034 | 0.8898 | PRSS7 | Enteropeptidase precursor (Enterokinase) |
| Ssc.27574.1.S1_at | -2.00 | 0.0040 | 0.8898 | LTBR | Tumor necrosis factor receptor superfamily member 3 precursor (Lymphotoxin-beta receptor) (Tumor necrosis factor receptor 2 related protein) (Tumor necrosis factor C receptor) |
| Ssc.6097.1.S1_a_at | -2.01 | 0.0040 | 0.8898 | TXNRD1 | Thioredoxin reductase 1, cytoplasmic precursor (TR) (TR1) |
| Ssc.494.1.S2_at | -2.27 | 0.0040 | 0.8898 | NP_940905 | Sodium- and chloride-activated ATP-sensitive potassium channel [Homo sapiens] |
| Ssc.15279.1.S1_at | -2.36 | 0.0043 | 0.8898 | Q9H5Y9 |  |
| Ssc.17063.1.A1_at | -2.36 | 0.0043 | 0.8898 | RB1 | Retinoblastoma-associated protein (PP110) (P105-RB) (RB) |
| Ssc.20986.1.S1_at | -2.19 | 0.0044 | 0.8898 | FKBP14 | FK506 binding protein 14 precursor (Peptidyl-prolyl cis- trans isomerase) (PPIase) (Rotamase) (22 kDa FK506-binding protein) (FKBP-22) |
| Ssc.25205.1.S1_at | -2.02 | 0.0045 | 0.8898 | Q6UWV2 |  |
| Ssc.8501.1.S1_at | 2.14 | 0.0050 | 0.8898 | ARL6IP5 | ADP-ribosylation-like factor 6 interacting protein 5; glutamate transporter EEAC1-associated protein; dermal papilla derived protein 11; putative MAPK activating protein PM27; PRA1 domain family 3; cytoskeleton related vitamin A responsive protein [Homo sapiens] |
| Ssc.9630.1.A1_at | -2.09 | 0.0055 | 0.8898 | CNKSR1 | Connector enhancer of kinase suppressor of ras 1 (Connector enhancer of KSR1) (hCNK1) (Connector enhancer of KSR-like) (CNK homolog protein 1) |
| Ssc.24265.1.S1_at | -2.05 | 0.0058 | 0.8898 | TNK1 | Tyrosine kinase, non-receptor, 1; tyrosine kinase non-receptor 1; tyrosine kinase non-receceptor 1 [Homo sapiens] |
| Ssc.16819.1.A1_at | 2.09 | 0.0059 | 0.8898 | ENC1 | Ectoderm-neural cortex-1 protein (ENC-1) (P53-induced protein 10) (Nuclear matrix protein NRP/B) |
| Ssc.7641.1.S1_at | 2.15 | 0.0060 | 0.8898 | Q5T7M9 |  |
| Ssc.24051.2.A1_at | -2.31 | 0.0061 | 0.8898 | ZZEF1 | Zinc finger, ZZ-type with EF hand domain 1 [Homo sapiens] |
| Ssc.4104.1.S1_at | 2.38 | 0.0064 | 0.8898 | DDIT4 | RTP801; HIF-1 responsive RTP801 [Homo sapiens] |
| Ssc.15638.1.A1_at | -2.67 | 0.0064 | 0.8898 | AMPD3 | AMP deaminase 3 (AMP deaminase isoform E) (Erythrocyte AMP deaminase) |
| Ssc.30181.1.A1_at | -2.02 | 0.0068 | 0.8898 | Q6ZRV2 |  |
| Ssc.25839.1.S1_at | -6.15 | 0.0070 | 0.8898 | ECE2 | 59 kDa protein |
| Ssc.17610.2.A1_at | -2.01 | 0.0072 | 0.8898 | Q8NAR6 | PREDICTED: hypothetical protein FLJ34907 |
| Ssc.4377.1.A1_at | -2.02 | 0.0074 | 0.8898 | TJP4 | Tight junction protein 4 (peripheral); protein incorporated later into tight junctions [Homo sapiens] |
| Ssc.494.1.S1_at | -2.17 | 0.0074 | 0.8898 | NP_940905 | Sodium- and chloride-activated ATP-sensitive potassium channel [Homo sapiens] |
| Ssc.16467.2.S1_at | -2.51 | 0.0078 | 0.8898 | ZBT7_HUMAN | Zinc finger and BTB domain containing protein 7 (Leukemia/lymphoma related factor) (Factor that binds to inducer of short transcripts protein 1) (Factor binding IST protein 1) (FBI-1) (HIV-1 1st-binding protein 1) (TTF-I interacting peptide 21) (TIP21) |
| Ssc.25149.1.S1_at | -2.21 | 0.0090 | 0.8898 | NP_690864 | Membrane-associated guanylate kinase-related 3 [Homo sapiens] |
| Ssc.2441.1.S1_at | -2.32 | 0.0091 | 0.8898 | SLC3A1 | Neutral and basic amino acid transport protein rBAT (B(0,+)-type amino acid transport protein) (NBAT) (D2H) |
| Ssc.1578.1.S1_at | 2.04 | 0.0092 | 0.8898 | GGA2 | ADP-ribosylation factor binding protein GGA2 (Golgi-localized, gamma ear-containing, ARF-binding protein 2) (Gamma-adaptin related protein 2) (Vear) (VHS domain and ear domain of gamma-adaptin) |
| Ssc.7176.1.A1_at | 3.91 | 0.0095 | 0.8898 | CXCR4 | C-X-C chemokine receptor type 4 (CXC-R4) (CXCR-4) (Stromal cell- derived factor 1 receptor) (SDF-1 receptor) (Fusin) (Leukocyte-derived seven transmembrane domain receptor) (LESTR) (LCR1) (FB22) (NPYRL) (HM89) (CD184 antigen) |
| Ssc.18359.1.S1_at | 3.61 | 0.0095 | 0.8898 | CCR1 | C-C chemokine receptor type 1 (C-C CKR-1) (CC-CKR-1) (CCR-1) (CCR1) (Macrophage inflammatory protein-1 alpha receptor) (MIP-1alpha-R) (RANTES-R) (HM145) (LD78 receptor) |
| Ssc.12329.2.S1_at | -2.37 | 0.0096 | 0.8898 | RGS5 | Regulator of G-protein signaling 5 (RGS5) |
| Ssc.20585.1.S1_at | 2.19 | 0.0099 | 0.8898 | GADD45G | Growth arrest and DNA-damage-inducible protein GADD45 gamma (Cytokine responsive protein CR6) |

**Additional file 2F**

| **Day 56 IN vs OUT** | | | | | |
| --- | --- | --- | --- | --- | --- |
| **affy.id** | **FC** | **P-value** | **Q-value** | **Gene Name** | **Product** |
| Ssc.4425.1.S1_at | 14.51 | 0.0001 | 0.3898 | PCDH15 | Protocadherin 15 precursor |
| Ssc.8960.1.A1_at | 3.37 | 0.0001 | 0.3898 | BPI | Bactericidal permeability-increasing protein precursor (BPI) (CAP 57) |
| Ssc.837.1.A1_at | 2.93 | 0.0001 | 0.3898 | BPI | Bactericidal permeability-increasing protein precursor (BPI) (CAP 57) |
| Ssc.30195.1.A1_at | 2.16 | 0.0001 | 0.3898 | NP_005790 | InaD-like protein isoform 3; protein associated to tight junctions; PDZ domain protein (Drosophila inaD-like); PALS1-associated tight junction protein; inactivation no after-potential D-like protein [Homo sapiens] |
| Ssc.23743.1.S1_at | -2.14 | 0.0001 | 0.3898 | MAPRE2 | Microtubule-associated protein, RP/EB family, member 2; T-cell activation protein, EB1 family; APC-binding protein EB1 [Homo sapiens] |
| Ssc.19640.1.A1_at | -2.31 | 0.0002 | 0.3898 | FCER1A | High affinity immunoglobulin epsilon receptor alpha-subunit precursor (FcERI) (IgE Fc receptor, alpha-subunit) (Fc-epsilon RI-alpha) |
| Ssc.2173.1.A1_at | 2.99 | 0.0005 | 0.5001 | ADR2_HUMAN | Adiponectin receptor protein 2 (Progestin and adipoQ receptor family member II) |
| Ssc.26321.1.S1_s_at | 2.69 | 0.0007 | 0.5001 | CYP2C18 | Cytochrome P450 2C18 (CYPIIC18) (P450-6B/29C) |
| Ssc.4069.1.S1_at | 2.00 | 0.0007 | 0.5001 | VRK3 | Serine/threonine-protein kinase VRK3 (Vaccinia-related kinase 3) |
| Ssc.6323.1.S1_at | 3.63 | 0.0009 | 0.5001 | ADFP | Adipophilin (Adipose differentiation-related protein) (ADRP) |
| Ssc.17905.1.A1_at | 2.26 | 0.0009 | 0.5001 | LRP1 | Low-density lipoprotein receptor-related protein 1 precursor (LRP) (Alpha-2-macroglobulin receptor) (A2MR) (Apolipoprotein E receptor) (APOER) (CD91) |
| Ssc.1402.1.A1_at | 2.14 | 0.0009 | 0.5001 | ADCK5 | AarF domain containing kinase 5 [Homo sapiens] |
| Ssc.8974.1.S1_at | 2.39 | 0.0010 | 0.5001 | CPT1A | Carnitine O-palmitoyltransferase I, mitochondrial liver isoform (CPT I) (CPTI-L) |
| Ssc.16013.1.S1_at | 2.16 | 0.0012 | 0.5001 | MMP1 | Interstitial collagenase precursor (Matrix metalloproteinase-1) (MMP-1) (Fibroblast collagenase) |
| Ssc.19681.1.S1_at | 2.03 | 0.0012 | 0.5001 | SLC23A1 | Solute carrier family 23, member 1 (Sodium-dependent vitamin C transporter 1) (hSVCT1) (Na(+)/L-ascorbic acid transporter 1) (Yolk sac permease-like molecule 3) |
| Ssc.7242.1.A1_at | -3.01 | 0.0012 | 0.5001 | STAU2 | Staufen homolog 2; staufen (Drosophila, RNA-binding protein) 2; staufen (Drosophila, RNA-binding protein) homolog 2 [Homo sapiens] |
| Ssc.26189.1.S1_a_at | 4.00 | 0.0013 | 0.5001 |  | 28 kDa protein |
| Ssc.15989.1.S1_at | 6.80 | 0.0015 | 0.5001 | PI3 | Elafin precursor (Elastase-specific inhibitor) (ESI) (Skin-derived antileukoproteinase) (SKALP) (WAP four-disulfide core domain protein 14) (Protease inhibitor WAP3) |
| Ssc.38.1.S1_at | 2.82 | 0.0017 | 0.5001 | TFRC | Transferrin receptor protein 1 (TfR1) (TR) (TfR) (Trfr) (CD71 antigen) (T9) (p90) |
| Ssc.26119.1.S1_at | 3.07 | 0.0018 | 0.5001 | HYAL1 | Hyaluronoglucosaminidase 1 isoform 1; hyaluronidase 1; tumor suppressor LUCA-1; plasma hyaluronidase [Homo sapiens] |
| Ssc.30724.1.S1_at | 2.82 | 0.0019 | 0.5001 | HERC6 | Hect domain and RLD 6 [Homo sapiens] |
| Ssc.12013.1.A1_at | 2.17 | 0.0019 | 0.5001 | SYTL4 | Synaptotagmin-like protein 4 (Exophilin 2) (Granuphilin) |
| Ssc.1492.2.A1_at | -2.01 | 0.0019 | 0.5001 | GNB5 | Guanine nucleotide-binding protein beta subunit 5 (Transducin beta chain 5) (Gbeta5) |
| Ssc.4804.1.S1_at | 2.67 | 0.0020 | 0.5001 | AGT | Angiotensinogen precursor [Contains: Angiotensin I (Ang I); Angiotensin II (Ang II); Angiotensin III (Ang III) (Des-Asp[1]- angiotensin II)] |
| Ssc.19212.1.S1_at | 2.41 | 0.0020 | 0.5001 | SLC25A20 | Mitochondrial carnitine/acylcarnitine carrier protein (Carnitine/acylcarnitine translocase) (CAC) |
| Ssc.24128.1.A1_at | -2.17 | 0.0020 | 0.5001 | O75458 | 20 kDa protein |
| Ssc.9536.1.A1_at | -2.04 | 0.0021 | 0.5001 | UGCGL2 | UDP-glucose:glycoprotein glucosyltransferase 2 precursor (UDP--Glc:glycoprotein glucosyltransferase 2) (UGT 2) (HUGT2) |
| Ssc.6514.3.S1_a_at | 3.74 | 0.0024 | 0.5001 | GALE | UDP-glucose 4-epimerase (EC 5.1.3.2) (Galactowaldenase) (UDP-galactose 4-epimerase) |
| Ssc.21339.1.S1_at | 2.50 | 0.0026 | 0.5001 | SSA1 | 52 kDa Ro protein (Sjogren syndrome type A antigen) (SS-A) (Ro(SS-A)) (52 kDa ribonucleoprotein autoantigen Ro/SS-A) |
| Ssc.12959.1.A1_at | 2.57 | 0.0027 | 0.5001 | ADAMTS12 | ADAMTS-12 precursor (A disintegrin and metalloproteinase with thrombospondin motifs 12) (ADAM-TS 12) (ADAM-TS12) |
| Ssc.16241.1.S1_at | 2.06 | 0.0027 | 0.5001 | PRKAR2A | cAMP-dependent protein kinase type II-alpha regulatory chain |
| Ssc.6514.1.S1_at | 2.48 | 0.0029 | 0.5001 | GALE | UDP-glucose 4-epimerase (EC 5.1.3.2) (Galactowaldenase) (UDP-galactose 4-epimerase) |
| Ssc.22075.3.A1_at | -2.43 | 0.0029 | 0.5001 | SELL | L-selectin precursor (Lymph node homing receptor) (Leukocyte adhesion molecule-1) (LAM-1) (Leukocyte surface antigen Leu-8) (TQ1) (gp90-MEL) (Leukocyte-endothelial cell adhesion molecule 1) (LECAM1) (CD62L) |
| Ssc.2487.2.S1_at | 2.33 | 0.0030 | 0.5001 | AMID | Apoptosis-inducing factor (AIF)-like mitchondrion-associated inducer of death; p53-responsive gene 3; apoptosis-inducing factor (AIF)-homologous mitochondrion-associated inducer of death; 5430437E11Rik [Homo sapiens] |
| Ssc.11108.1.A1_at | -2.49 | 0.0033 | 0.5001 | PMP22 | Peripheral myelin protein 22 (PMP-22) |
| Ssc.27512.1.A1_at | 2.09 | 0.0034 | 0.5001 | RNF123 | Ring finger protein 123 [Homo sapiens] |
| Ssc.21108.1.S1_at | 2.05 | 0.0035 | 0.5001 | C5 | Complement C5 precursor [Contains: C5a anaphylatoxin] |
| Ssc.9772.1.S1_at | -2.38 | 0.0035 | 0.5001 | RSBN1L | Round spermatid basic protein 1-like [Homo sapiens] |
| Ssc.2202.2.S1_at | 2.37 | 0.0036 | 0.5021 | PREB | Prolactin regulatory element-binding protein |
| Ssc.27596.1.S1_at | 2.40 | 0.0037 | 0.5021 | AQP1 | Aquaporin-CHIP (Water channel protein for red blood cells and kidney proximal tubule) (Aquaporin 1) (AQP-1) (Urine water channel) |
| Ssc.4892.1.S1_at | 2.12 | 0.0039 | 0.5067 | COL18A1 | Collagen alpha 1(XVIII) chain precursor [Contains: Endostatin] |
| Ssc.12756.1.A1_at | 2.31 | 0.0040 | 0.5107 | Q8IV60 |  |
| Ssc.86.1.S1_at | 2.42 | 0.0041 | 0.5107 | DHRS4 | Dehydrogenase/reductase SDR family member 4 (NADPH- dependent carbonyl reductase/NADP-retinol dehydrogenase) (CR) (PHCR) (Peroxisomal short-chain alcohol dehydrogenase) (NADPH-dependent retinol dehydrogenase/reductase) (NDRD) (SCAD-SRL) (humNRDR) (PSCD) |
| Ssc.7558.1.A1_at | 2.06 | 0.0042 | 0.5107 | RAB3C | Ras-related protein Rab-3C |
| Ssc.16227.1.S1_at | 3.87 | 0.0048 | 0.5120 | ZDHHC9 | Zinc finger DHHC domain containing protein 9 (Zinc finger protein 379) (CGI-89) (UNQ261/PRO298) |
| Ssc.10932.1.S1_at | 2.00 | 0.0048 | 0.5120 | PECI | Peroxisomal 3,2-trans-enoyl-CoA isomerase (Dodecenoyl-CoA delta-isomerase) (D3,D2-enoyl-CoA isomerase) (DBI-related protein 1) (DRS-1) (Hepatocellular carcinoma-associated antigen 88) |
| Ssc.5407.1.A1_at | -2.45 | 0.0048 | 0.5120 | Q9H6L5 |  |
| Ssc.28316.1.S1_at | 2.09 | 0.0050 | 0.5142 | Q7Z6I9 |  |
| Ssc.12788.1.S1_at | 2.05 | 0.0053 | 0.5260 | RNF121 | Ring finger protein 121 isoform 1 [Homo sapiens]. |
| Ssc.1031.1.S1_at | 5.52 | 0.0061 | 0.5367 | OAS1 | 2'-5'-oligoadenylate synthetase 1 ((2-5')oligo(A) synthetase 1) (2-5A synthetase 1) (p46/p42 OAS) (E18/E16) |
| Ssc.20783.1.S1_at | 2.40 | 0.0062 | 0.5367 | Q8NB46 |  |
| Ssc.27896.1.S1_at | 2.02 | 0.0063 | 0.5367 | SIAT8D | CMP-N-acetylneuraminate-poly-alpha-2,8-sialyl transferase (Alpha-2,8-sialyltransferase 8D) (ST8Sia IV) (Polysialyltransferase-1) |
| Ssc.2680.1.S1_at | 2.01 | 0.0067 | 0.5367 | Q96AG4 |  |
| Ssc.26005.1.S1_at | 4.48 | 0.0069 | 0.5367 | ZBP1 | Z-DNA binding protein 1 (Tumor stroma and activated macrophage protei DLM-1) |
| Ssc.19393.1.S1_at | 4.33 | 0.0075 | 0.5367 | DMBT1 | Deleted in malignant brain tumors 1 isoform b precursor [Homo sapiens] |
| Ssc.10993.1.S1_a_at | 2.62 | 0.0075 | 0.5367 | LGALS9 | Galectin-9 (HOM-HD-21) (Ecalectin) |
| Ssc.8359.2.A1_at | 2.20 | 0.0075 | 0.5367 | SEMA6A | Semaphorin 6A precursor (Semaphorin VIA) (Sema VIA) (Semaphorin 6A-1) (SEMA6A-1) |
| Ssc.6028.1.S1_at | -2.68 | 0.0078 | 0.5367 | Q8TAD7 |  |
| Ssc.19659.2.S1_at | 2.50 | 0.0079 | 0.5367 | NP_005759 | Gene rich cluster, C3f gene [Homo sapiens] |
| Ssc.30737.1.A1_s_at | 2.45 | 0.0082 | 0.5367 | PNPT1 | Polyribonucleotide nucleotidyltransferase 1; polynucleotide phosphorylase-like; 3'-5' RNA exonuclease [Homo sapiens]. |
| Ssc.29676.1.A1_a_at | 2.25 | 0.0082 | 0.5367 | COBLL1 | COBL-like 1 [Homo sapiens] |
| Ssc.21987.2.S1_at | 2.23 | 0.0082 | 0.5367 | IFRD1 | Interferon-related developmental regulator 1 (Nerve growth factor- inducible protein PC4) |
| Ssc.9957.1.A1_at | 2.44 | 0.0085 | 0.5371 | CCL8 | Small inducible cytokine A8 precursor (CCL8) (Monocyte chemotactic protein 2) (MCP-2) (Monocyte chemoattractant protein 2) (HC14) [Contains: MCP-2(6-76)] |
| Ssc.21.1.S1_s_at | 5.36 | 0.0086 | 0.5371 | DDX58 | DEAD/H (Asp-Glu-Ala-Asp/His) box polypeptide RIG-I; RNA helicase; DEAD/H (Asp-Glu-Ala-Asp/His) box polypeptide [Homo sapiens] |
| Ssc.5704.1.S1_at | 2.32 | 0.0091 | 0.5371 | TNA | Tetranectin precursor (TN) (Plasminogen-kringle 4 binding protein) |
| Ssc.6528.1.S1_at | 2.06 | 0.0092 | 0.5371 | AGPAT2 | 1-acyl-sn-glycerol-3-phosphate acyltransferase beta (1- AGP acyltransferase 2) (1-AGPAT 2) (Lysophosphatidic acid acyltransferase-beta) (LPAAT-beta) (1-acylglycerol-3-phosphate O- acyltransferase 2) |
| Ssc.22620.1.S1_at | 8.32 | 0.0093 | 0.5371 | IFIT2 | Interferon-induced protein with tetratricopeptide repeats 2 (IFIT-2) (Interferon-induced 54 kDa protein) (IFI-54K) (ISG-54 K) |
| Ssc.670.2.S1_at | 6.92 | 0.0093 | 0.5371 | LYZ | Lysozyme C precursor (1,4-beta-N-acetylmuramidase C) |
| Ssc.21669.1.S1_at | 2.18 | 0.0094 | 0.5371 | ACADVL | Acyl-CoA dehydrogenase, very-long-chain specific, mitochondrial precursor (VLCAD) |
| Ssc.21548.1.S1_at | 2.15 | 0.0094 | 0.5371 | KDELR3 | ER lumen protein retaining receptor 3 (KDEL receptor 3) |
| Ssc.25840.1.S1_at | 2.40 | 0.0097 | 0.5371 | EHF | Ets homologous factor; epithelium-specific ets factor 3 [Homo sapiens] |
