# Supplementary material for: Environmentally-acquired bacteria influence microbial diversity and natural innate immune responses at gut surfaces
Source: BMC Biol. 2009 Nov 20;7:79. doi: 10.1186/1741-7007-7-79 (PMC2785767; doi:10.1186/1741-7007-7-79)
Supplement: Additional file 3 — Real-time PCR primer sequences. Porcine gene-specific primers were designed for the sequence of interest using Primer Express Software v3.0 (Applied Biosystems). [file 1741-7007-7-79-S3.DOC]

**Additional file 3** - **Real-time PCR primer sequences**

| **Gene** | **Forward Primer** | **Reverse Primer** |
| --- | --- | --- |
| *EEF1A1* | CCTGGCAAGCCCATGTGT | TGTCTCATGTCACGAACAGCAA |
| *CCL28* | TGCAGCACACAGGACTTGCT | GGAGGCAATGGGAAGTATAGCTT |
| *CCL8* | AAGACCAAAGCCGACAAGGA | TCATGGAATTCTGGACCCACTT |
| *CXCL12* | TCCTGCCATGGGTGTCAAG | AACGTGGCTCTCAAAGAATCG |
| *CXCL9* | ATTTGCCCCAAGCCCTTCT | GCTGACCTGTTTCTCCCACTCT |
| *CXCR4* | CATCTTCTTAACTGGCATAGTGGGTAA | CCGTCATGCTTCTCAGTTTCTTC |
| *IFIT2* | CCTATCTCGCCTGCCTCTGT | TGGAGCACTTGTTTGGCTACA |
| *FKBP5* | GTAGCTTCCCCCCAAACCA | TGAAATGAGCTGGACTTAAACTGTTG |
| *IRF7* | CTGCGATGGCTGGATGAAG | TAAAGATGCGCGAGTCGGA |
| *IRP6* | CAATCGCTTCAATGTGGAAGAAG | CCCTCAATTAAGAGGCATTGGA |
| *MT1J* | GTGTAGCTGCTGTGCCTGATG | CAGGTTGGCCCACATTCC |
| *MX* | GGCGTGGGAATCAGTCATG | AGGAAGGTCTATGAGGGTCAGATCT |
| *PDK4* | TGGTATGTGTGAAAGCAAAGGAA | AAGTGGGCCTGAACAGTCAGTT |
| *SELL* | CGCTCCGTCTGTTCCCATT | TGAACTAATAAAGACTGCCAACTGAAG |
| *PI3* | CCTGCCCCAGGATTCTGA | TGAGCATCACTCAAACACCTGTT |
| *SQLE* | TTCGTCTCTCTGGCCAACAA | CCCCAGCGACAGGAATACC |
| *TFRC* | ATCACTTCCTTTCACCCTATGTATCTC | GCCAGAGCCCCAGAAGATATG |

Porcine gene-specific primers were designed for the sequence of interest using Primer Express Software v3.0.
